# The transcription factor ZEB2 mediates the antitumor efficacy of tumor-infiltrating lymphocytes in non–small cell lung cancer

**DOI:** 10.1038/s41419-025-08112-y

**Published:** 2025-11-07

**Authors:** Jiajia Wang, Fei Liu, Yongyong Li, Jiaojiao Gao, Shasha Yang, Mei Tian, Lili Deng, Yan Yang, Beilei Gong, Chengling Zhao, Huiyuan Gong, Zongyu Xie, Yongchun Zhou, Rongzhong Huang, Qiang Luo, Depeng Jiang, Xiaojing Wang

**Affiliations:** 1 Anhui Province Key Laboratory of Respiratory Tumor and Infectious Disease, the Department of Pulmonary Critical Care Medicine, First Affiliated Hospital of Bengbu Medical University, Bengbu, China; 2 Precision Medicine Center, the Second Affiliated Hospital of Chongqing Medical University, Chongqing, China; 3 Molecular Diagnosis Center, Joint Research Center for Regional Diseases of Institute of Health and Medicine (IHM), the First Affiliated Hospital of Bengbu Medical University, Bengbu, China; 4 Department of Geriatric Medicine, the Second Affiliated Hospital of Chongqing Medical University, Chongqing, China; 5 Anhui Province Key Laboratory of Respiratory Tumor and Infectious Disease, Department of Medical Oncology, the First Affiliated Hospital of Bengbu Medical University, Bengbu, China; 6 Anhui Province Key Laboratory of Respiratory Tumor and Infectious Disease, Department of Thoracic Surgery, the First Affiliated Hospital of Bengbu Medical University, Bengbu, China; 7 Anhui Province Key Laboratory of Respiratory Tumor and Infectious Disease, Department of Radiology, the First Affiliated Hospital of Bengbu Medical University, Bengbu, China; 8 Anhui Province Key Laboratory of Respiratory Tumor and Infectious Disease, Department of Radiation Oncology, the First Affiliated Hospital of Bengbu Medical University, Bengbu, China; 9 Key Laboratory of Molecular Biology for Infectious Diseases (Ministry of Education), Institute for Viral Hepatitis, Department of Infectious Diseases, The Second Affiliated Hospital, Chongqing Medical University, Chongqing, China; 10 Department of Respiratory Medicine, the Second Affiliated Hospital of Chongqing Medical University, Chongqing, China

**Keywords:** Cancer microenvironment, Cancer

## Abstract

Immune checkpoint blockade (ICB) offers an in vivo approach to activate CD8^+^ tumor-infiltrating lymphocytes (CD8^+^TILs) in cases of advanced non–small cell lung cancer (NSCLC). A large fraction of NSCLC patients is unresponsive to ICBs and relapse due to the development of dysfunctional CD8^+^TILs with impaired cytotoxicity. Therefore, an improved understanding of regulator(s) that favor the development of cytotoxic T_eff_ cells over dysfunctional CD8^+^TILs is required for the success of ICB therapy in NSCLC patients. Here, our metaVIPER-based scRNA-seq analysis of deep CD8^+^ cell scRNA-seq data from 14 treatment-naïve NSCLC patients revealed that the master regulon ZEB2 may drive CD8^+^ differentiation along the cytotoxic effector trajectory in NSCLC tumors. In vitro, ZEB2 acts downstream of T-bet to stimulate lung tumor-reactive T_eff_ cell differentiation. This T-bet/ZEB2 axis displays immunotherapeutic effects on KP.SIY lung tumors independent of ICB therapy and mediates the therapeutic effects of murine serum albumin-fused IL-2 + IL-12 combination immunotherapy (IL2-MSA + IL12-MSA) in mice. IL2-MSA + IL12-MSA operates through a parallel STAT4/FOXO1-mediated mechanism that promotes CD8^+^TIL T-bet/ZEB2 expression and lung tumor-reactive T_eff_ cell differentiation. In conclusion, immunotherapeutic regimens that support ZEB2 activity in CD8^+^ cells may show promise in NSCLC patients.

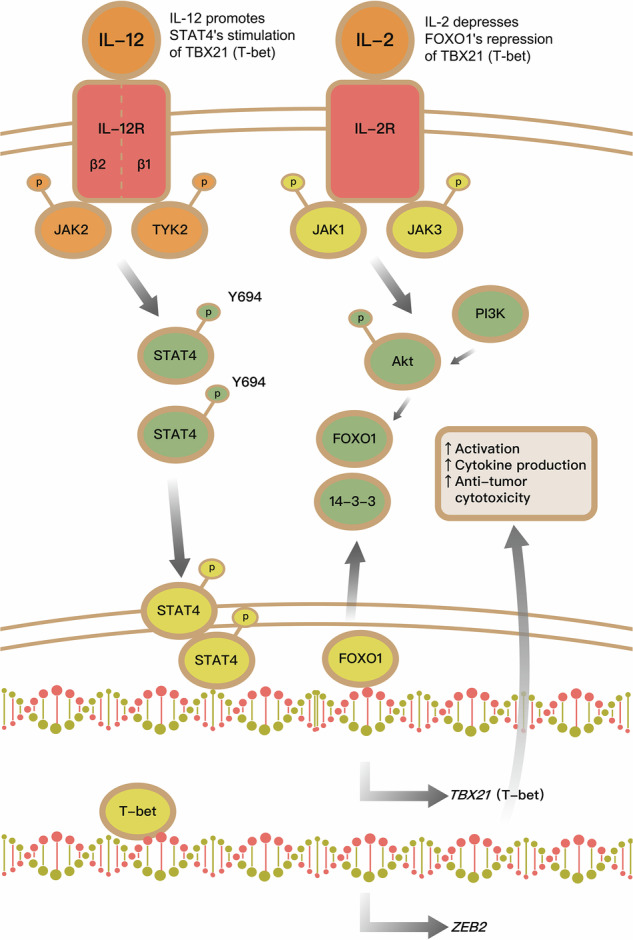

## Introduction

Tumor infiltration by lymphocytes is associated with a favorable survival prognosis [1] and a better clinical response to immune checkpoint blockade (ICBs) [2]. ICBs are thought to “reprogram” CD8^+^ tumor-infiltrating lymphocytes (CD8^+^TILs) to produce antitumor responses by targeting inhibitory receptors such as programmed cell death protein 1 (PD-1), which are highly expressed by most CD8^+^TIL populations [3, 4]. The success of ICBs has established the importance of cancer immunotherapy, where it has been adopted as one of the standards of care for advanced non–small cell lung cancer (NSCLC). ICB therapy offers an in vivo approach to activate tumor-specific CD8^+^TILs, albeit in some cases, this modality does not create a sufficiently robust anti-tumor response. Therefore, a large fraction of NSCLC patients does not respond to ICBs and relapse.

Kissick et al. and Amigorena et al., with support from a number of scRNA-seq studies, propose a multi-step activation model as the best explanation for how CD8^+^TILs respond to cancer [5–10]: (i) undifferentiated CD8^+^TIL precursors proliferate in tumor-draining lymph nodes but fail to express effector molecules; (ii) these TIL precursors turn on co-stimulatory receptors and several chemokine receptors and slowly migrate through the juxtatumor tissue bearing markers of memory-resident T-cells (i.e., juxtatumor tissue-resident, memory-like CD8^+^TIL precursors [T_rms_]); and (iii) only once in the tumor do tumor-resident effector memory CD8^+^TILs (T_ems_) receive additional co-stimulation from antigen-presenting cells and transition into terminally-differentiated tumor-resident effector CD8^+^TILs (T_eff_). The presence of a T-cell-derived IFN-γ signature (and its target PD-L1) is predictive for ICB response in NSCLC [9, 11]. Fully-differentiated T_eff_ (and a subset of CX3CR1^+^T_ems_) express IFN-γ and exert cytotoxic activity in patients with lung cancer [8, 12, 13]. In contrast, tumor-resident, terminally-exhausted CD8^+^TILs (T_exh_) and lung cancer–specific dysfunctional CD8^+^TILs (T_Ldys_) are dysfunctional TILs characterized by impaired cytotoxicity, failure to make IFN-γ, and a strong association with non-response to ICB therapy in lung cancer patients [8, 9]. In fact, KP lung tumor-reactive CD8^+^TILs exhibit an ICB-refractory T_Ldys_ phenotype; IL2 + IL12 combination immunotherapy restores KP lung tumor-reactive T_eff_ cell differentiation that suppresses KP lung tumor growth in mice [9, 14]. This evidence emphasizes a critical role of differentiation favoring T_eff_ cells over T_exh_ and T_Ldys_ cells for the success of ICB therapy in NSCLC patients.

To identify master regulon(s) that drive T_eff_ cell differentiation, we can employ the metaVIPER algorithm, which leverages highly multiplexed, tissue-specific gene-reporter assays to accurately measure the activity of up to ∼6500 regulatory proteins (regulons) on a single-cell basis, including transcription factors (TFs), co-factors (co-TFs), signaling proteins, and surface markers, based on the expression of their downstream regulatory targets [15]. Single-cell, tissue-specific regulons are inferred using ARACNe, an information theoretic algorithm that has been experimentally validated in multiple tissue contexts, with a > 70% accuracy in target identification [16]. We used deep CD8^+^ cell single-cell RNA sequencing (scRNA-seq) data from lung tumor, adjacent normal tissues, and peripheral blood from 14 treatment-naïve NSCLC patients. We developed a metaVIPER-based scRNA-seq analysis pipeline to assess single-cell protein activity from single-cell ARACNe networks, followed by an optimized single-cell clustering approach. Consistent with viral disease models [17, 18], this analysis revealed that the master regulon ZEB2 may drive the differentiation of T_eff_ cells in NSCLC tumors. We further demonstrate that ZEB2 acts downstream of the transcription factor TBX21 (T-bet) to stimulate lung tumor-reactive T_eff_ cell differentiation in vitro. We show that this T-bet/ZEB2 axis in CD8^+^TILs displays immunotherapeutic effects on SIYRYYGL antigen-positive *Kras*^G12D/+^;*p53*^fl/fl^ (KP.SIY) lung tumors independent of ICB therapy and is the primary mediator of the immunotherapeutic effects of murine serum albumin-fused IL-2 + IL-12 (IL2-MSA + IL12-MSA) on KP.SIY lung tumors. We finally demonstrate that IL2-MSA + IL12-MSA combination immunotherapy operates through a parallel STAT4/FOXO1-mediated mechanism that promotes CD8^+^TIL T-bet/ZEB2 expression and lung tumor-reactive T_eff_ cell differentiation.

## Results

### metaVIPER-based scRNA-seq analysis reveals that the master regulon ZEB2 may drive CD8^+^ differentiation along the cytotoxic effector trajectory in NSCLC tumors

**Fig. 1:**
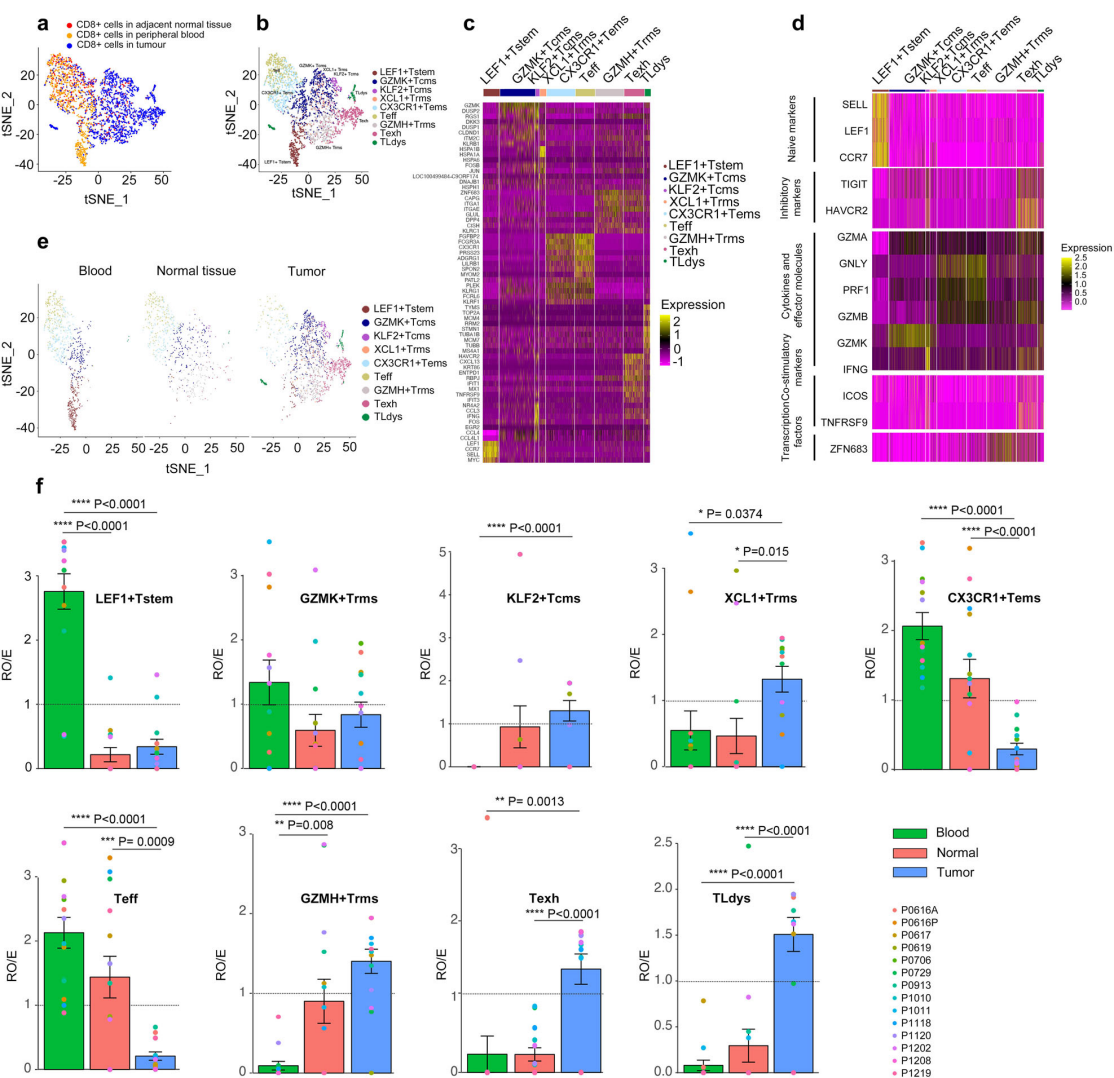
Deep scRNA-seq analyses of CD8^+^ T-cells from treatment-naïve NSCLC patients. **a** t-SNE projection of CD8^+^ cells from the 14 treatment-naïve NSCLC patients. Each dot represents one single cell and is color-coded by tissue type. **b** t-SNE projection of CD8^+^ cells from the 14 treatment-naïve NSCLC patients displaying the nine main CD8^+^ clusters. Each dot represents one single cell and is color-coded by cell cluster. **c** Expression heatmap of the signature genes for the nine main CD8^+^ clusters. **d** Expression heatmap of key T-cell marker genes across the nine main CD8^+^ clusters. **e** t-SNE projection of CD8^+^ cells from the 14 treatment-naïve NSCLC patients displaying the nine main CD8^+^ clusters analyzed by tissue type. Each dot represents one single cell and is color-coded by cell cluster. **f** Tissue preference for the nine main CD8^+^ clusters. Each chart displays one cell cluster and is color-coded by tissue type (bars) and patient (dots). The ratio of observed cell numbers:random expectation (R_O/E_) was used for adjusting cell-sampling biases. R_O/E_ > 1 shows enrichment. *P*-values are displayed [one-way ANOVA].

Guo et al. has published a scRNA-seq dataset derived from fourteen treatment-naïve NSCLC patients consisting of eleven lung adenocarcinoma patients and three squamous cell carcinoma patients [8]. Deep scRNA-seq analyses were performed on CD8^+^ cells across three tissue compartments: peripheral blood, NSCLC tumors, and adjacent normal lung tissues (Fig. 1a). To elucidate CD8^+^ cell heterogeneity, deep scRNA-seq clustering analysis based on t-distributed stochastic neighbor embedding (t-SNE) [19] identified nine distinct CD8^+^ clusters across a total of 3805 CD8^+^ cells (Fig. 1b and Supp. Fig. S1a). The expression of signature genes (Supp. File S1) and known functional markers supported the distinct identities and phenotypes (e.g., stem-cell like naïve, resident memory, effector, exhausted, etc.) of the nine CD8^+^ clusters (Fig. 1c, d and Supp. Fig. S1b). As expected, the two effector clusters (CX3CR1^+^T_ems_ and T_eff_) were characterized by elevated expression of the cytotoxicity markers GNLY, GZMB, and PRF1 [20] and decreased expression of the inhibitory markers HAVCR2 and TIGIT [21]. The nine CD8^+^ clusters displayed varied tissue distributions (Fig. 1e, f). Specifically, the stem-cell-like naïve LEF1^+^T_stem_ cluster was primarily localized in the circulation (R_O/E_ > 2.5), and the two effector CD8^+^ cell clusters (CX3CR1^+^T_ems_ and T_eff_) were primarily localized in the circulation and adjacent normal lung tissue (R_O/E_ > 1). The exhausted T_exh_ cluster and dysfunctional T_Ldys_ cluster were primarily localized in lung tumors (R_O/E_ > 1), which is consistent with previous studies supporting the development of exhausted and dysfunctional CD8^+^TIL phenotypes in lung tumors [5–9].

**Fig. 2:**
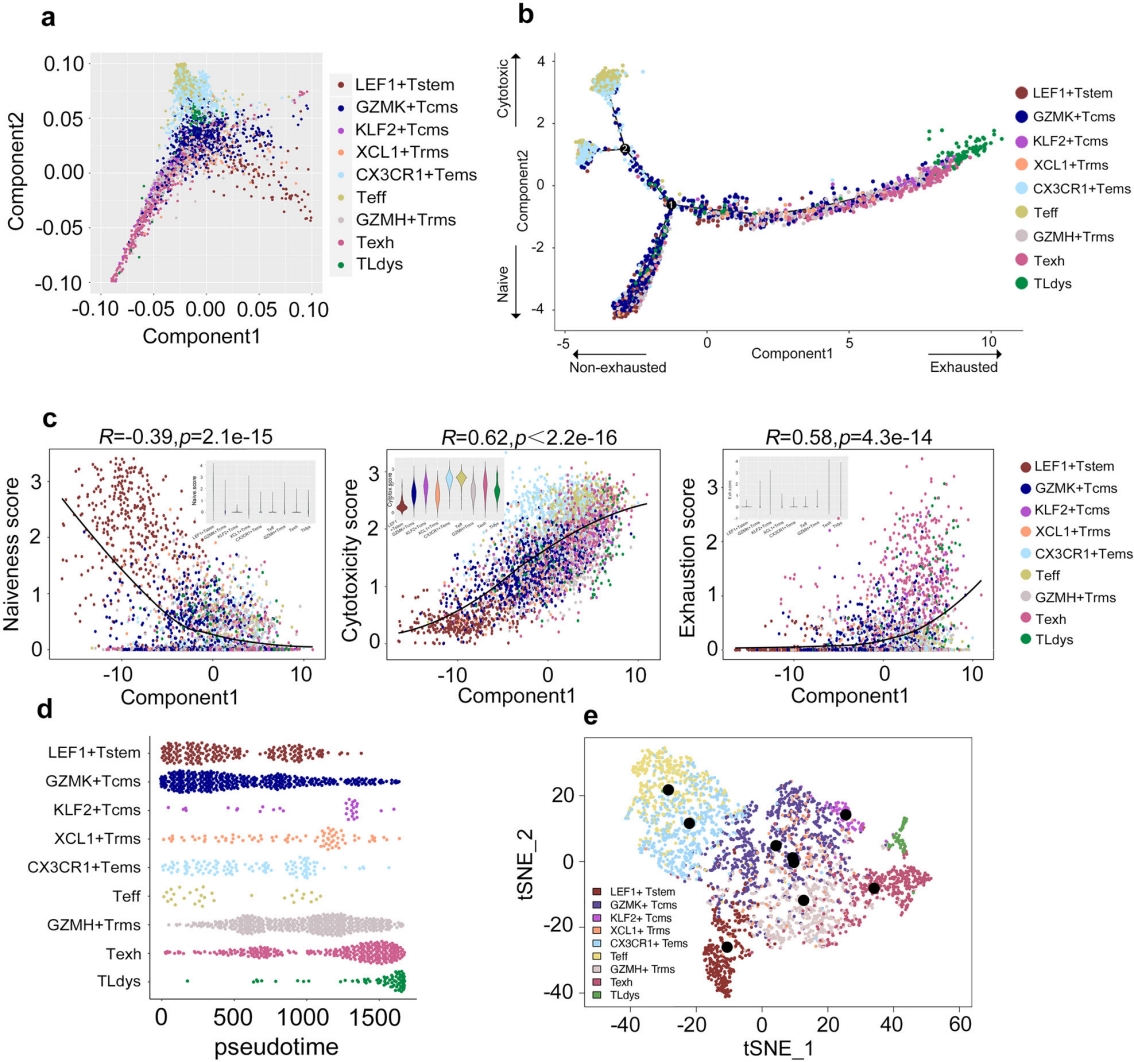
Computational inference of the developmental trajectories of the CD8^+^ clusters. **a** Monocle-based inference displays the nine main CD8^+^ clusters and **b** the branched developmental trajectory of CD8^+^ cells across two dimensions (naiveness and exhaustion). Each dot represents one single cell and is color-coded by cell cluster. **c** Correlation plots of the Monocle components with scores for naiveness, cytotoxicity, and exhaustion. Solid black lines depict the LOESS fits between the Monocle components and the respective scores. Embedded violin plots display the distributions of respective scores by cell cluster. *R* and *P*-values are displayed [Pearson correlation]. **d** Pseudotime analysis by cell cluster and **e** t-SNE projection of CD8^+^ cells displaying the branched developmental trajectory of CD8^+^ cells with a LEF1^+^T_stem_ root (A), an intermediate T_cms_/T_rms_ trunk (B1/B2/C), a cytotoxic effector branch (CX3CR1^+^T_ems_→T_eff_) (D1 → D2), a distinct exhausted (T_exh_) branch (E1 → E2.1), and a distinct dysfunctional (T_Ldys_) branch (E1 → E2.2). Each dot represents one single cell and is color-coded by cell cluster.

Kissick and Amigorena’s multi-step activation model proposes that CD8^+^ cells transition across phenotypes as they progress from the circulation through normal lung tissue and finally infiltrate the lung tumor [6, 10]. To characterize these proposed phenotypic transitions, the developmental trajectories of the nine CD8^+^ clusters were computationally inferred based on gene expression data inputted into the unsupervised inference algorithm Monocle [22]. Monocle inferred a root-trunk-branch structure, stemming from a LEF1^+^T_stem_ root that transitions into an intermediate T_cms_/T_rms_ trunk which branches out into a CX3CR1^+^T_ems_→T_eff_ branch and a T_exh_ branch (Fig. 2a). By applying naïveness, cytotoxicity, and exhaustion scores calculated from previously-established gene signatures [8] to the nine CD8^+^ clusters, we confirmed that the naïve, non-exhausted phenotypes (LEF1^+^T_stem_, T_cms_/T_rms_) branch out into a cytotoxic effector trajectory (CX3CR1^+^T_ems_→T_eff_) and an exhausted/dysfunctional trajectory (populated by T_exh_ and T_Ldys_ cells) (Fig. 2b and Supp. Fig. S2). We confirmed that Component 2 of the Monocle trajectory (*y*-axis) was negatively correlated with the naïveness score and positively correlated with the cytotoxicity score; moreover, Component 1 of the Monocle trajectory (*x*-axis) was positively correlated with exhaustion (Fig. 2c and Supp. File S2). Applying Monocle pseudotime analysis (Fig. 2d), we further clarified our proposed root-trunk-branch structure with a LEF1^+^T_stem_ root, an intermediate T_cms_/T_rms_ trunk, a cytotoxic effector branch (CX3CR1^+^T_ems_→T_eff_), a distinct exhausted (T_exh_) branch, and a distinct dysfunctional (T_Ldys_) branch (Fig. 2e). In sum, our findings suggest that naïve, non-exhausted CD8^+^ cells transition into (i) a cytotoxic effector trajectory (i.e., CX3CR1^+^T_ems_→T_eff_ cells) primarily localized in the circulation and adjacent normal lung tissue, (ii) an exhausted (T_exh_) branch primarily localized within lung tumors, or (iii) a dysfunctional (T_Ldys_) branch primarily localized within lung tumors.

**Fig. 3:**
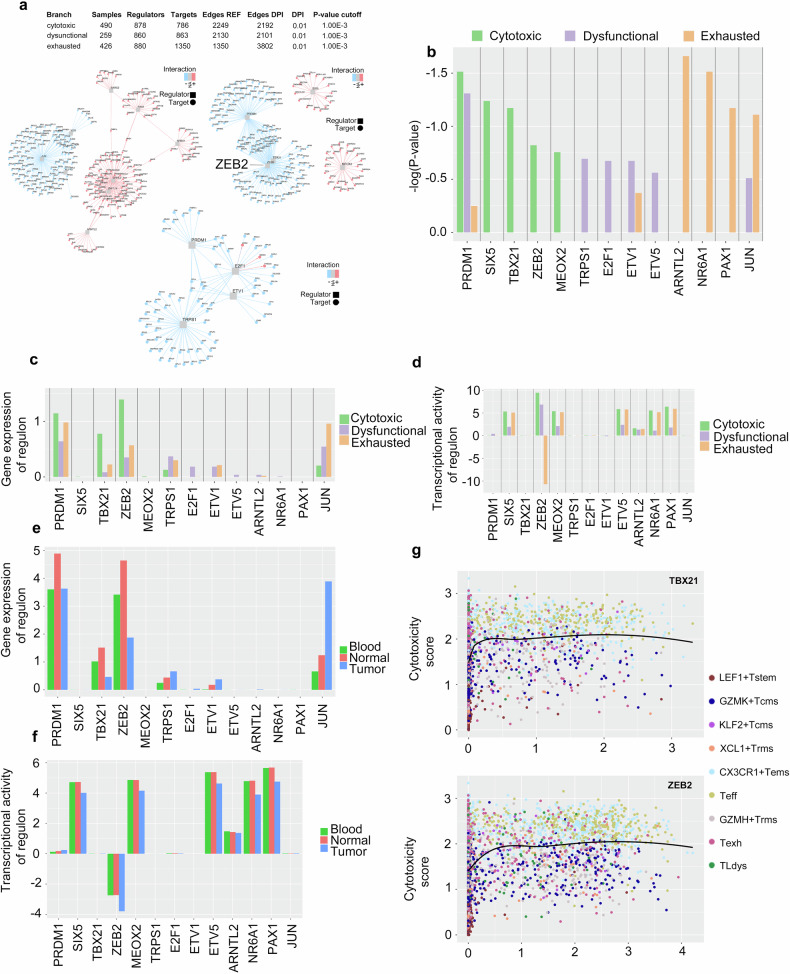
Identification of master regulon(s) that may contribute to CD8^+^ differentiation along the cytotoxic effector trajectory. **a** Visualization of the metaVIPER-based gene regulatory networks (GRNs) for the cytotoxic effector branch (CX3CR1^+^T_ems_→T_eff_ cells), the dysfunctional (T_Ldys_) branch, and the exhausted (T_exh_) branch. The grey square nodes indicate the core transcription factors (regulons), while the circular nodes indicate their target genes (colored by mode of regulation). **b** Plot of the top-ranking metaVIPER-based regulons for each developmental branch. **c** Gene expression levels and **d** metaVIPER-based transcriptional activity levels for the top-ranking regulons for each developmental branch. **e** Gene expression levels and **f** metaVIPER-based transcriptional activity levels for the top-ranking regulons by tissue type. **g** Correlation plots of ZEB2 and TBX21 gene expression levels with cytotoxicity scores. Solid black lines depict the LOESS fits between the gene expression levels and the cytotoxicity scores.

To identify master regulon(s) that may contribute to CD8^+^ differentiation along the cytotoxic effector trajectory (CX3CR1^+^T_ems_→T_eff_ cells) in NSCLC tumors, we applied metaVIPER-based scRNA-seq analysis to construct GRNs of the cytotoxic effector branch (CX3CR1^+^T_ems_→T_eff_ cells), the dysfunctional (T_Ldys_) branch, and the exhausted (T_exh_) branch (Fig. 3a). From these GRNs, we established a set of criteria to identify TFs that display a high statistical significance in regulating the defined signature genes (Fig. 3b and Supp. Fig. S3). We identified the pro-effector TFs ZEB2, TBX21 (T-bet), and PRDM1 (Blimp-1) [23] as three core master regulons in the cytotoxic effector GRN. Interestingly, PRDM1 was also identified as a core master regulon in the dysfunctional GRN, suggesting a conflicting role for PRDM1 in contributing to the cytotoxic effector phenotype in NSCLC. Although the gene expression levels of ZEB2 and TBX21 were elevated in the cytotoxic effector trajectory relative to the dysfunctional and exhausted branches (Fig. 3c), the metaVIPER-derived transcriptional activity of ZEB2 was elevated in the cytotoxic effector trajectory with a moderate declination in the dysfunctional branch and a pronounced declination in the exhausted branch (Fig. 3d). Analyzing by tissue distribution, the gene expression levels of ZEB2 and TBX21 (Fig. 3e) as well as ZEB2 transcriptional activity (Fig. 3f) were downregulated in the tumor environment relative to the adjacent normal tissue and peripheral blood. We confirmed that ZEB2 and TBX21 displayed strong positive correlations with the cytotoxicity score at low gene expression levels, which tapers off as gene expression levels increase (Fig. 3g). This analysis suggests that ZEB2 may be a key driver of CD8^+^ differentiation along the cytotoxic effector trajectory in NSCLC tumors.

### Zeb2 stimulates lung tumor-reactive Teff cell differentiation

**Fig. 4:**
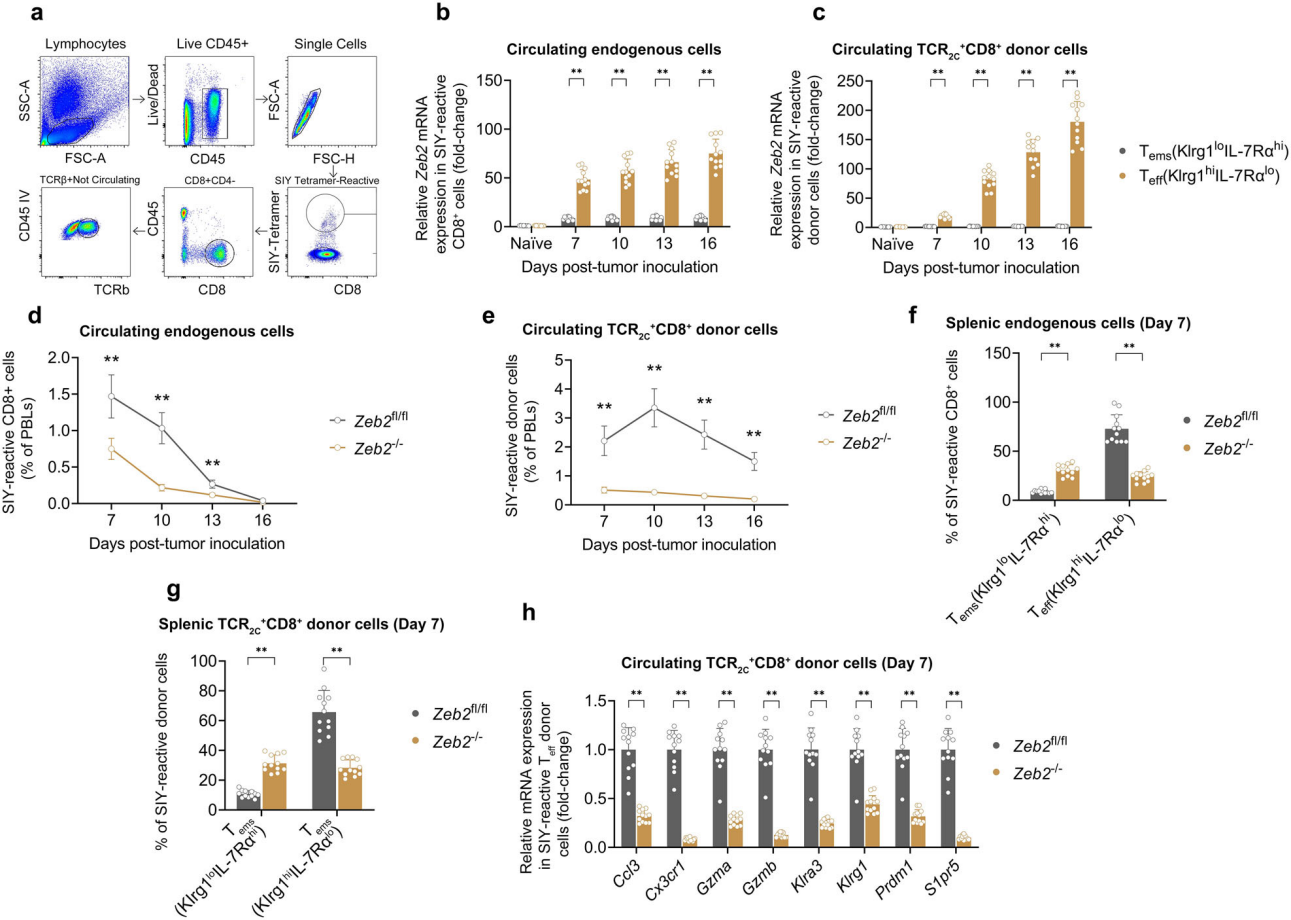
Zeb2 stimulates lung tumor-reactive T_eff_ cell differentiation. **a** Representative images showing the gating strategy to isolate circulating SIY-reactive CD8^+^ cells. **b**, **c** qPCR analysis of *Zeb2* mRNA expression in purified naïve CD8^+^ cells (CD44^lo^CD62L^hi^), SIY-reactive T_eff_ CD8^+^ cells (Klrg1^hi^IL-7Rα^lo^), or SIY-reactive T_ems_ CD8^+^ cells (Klrg1^lo^IL-7Rα^hi^) on the indicated days post-tumor inoculation in WT mice using the (**b**) endogenous CD8^+^ model and the (**c**) TCR_2C_^+^CD8^+^ adoptive transfer model. **d**, **e** The percentage of SIY-reactive CD8^+^ cells on the indicated days post-tumor inoculation in *Zeb2*-floxed (*Zeb2*^fl/fl^) mice and *Zeb2*^fl/fl^;*Gzmb*-Cre (*Zeb2*^−/−^) mice using the (**d**) endogenous CD8^+^ model and the (**e**) TCR_2C_^+^CD8^+^ adoptive transfer model. **f**, **g** The percentage of SIY-reactive CD8^+^ subsets (T_eff_, T_ems_) on day 7 post-tumor inoculation in *Zeb2*^fl/fl^ mice and *Zeb2*^−/−^ mice using the (**f**) endogenous CD8^+^ model and the (**g**) TCR_2C_^+^CD8^+^ adoptive transfer model. **h** qPCR analysis of T_eff_-signature genes in SIY-reactive T_eff_ cells on day 7 post-tumor inoculation in *Zeb2*^fl/fl^ mice and *Zeb2*^−/−^ mice using the TCR_2C_^+^CD8^+^ adoptive transfer model. Data depicts means ± SDs from *n* = 12 mice/group. *P < 0.05; **P < 0.01 [**a–g** two-way ANOVA, **h** *t*-test].

To analyze *Zeb2* gene expression in lung tumor-reactive T_eff_ cells, we used the murine KP.SIY lung tumor model [14], in which the lung tumor neoepitope antigen SIYRYYGL (SIY) is presented to TCR_2C_^+^CD8^+^ cells via the MHC class I protein H2-K^b^ [24, 25]. In our first KP.SIY model, we were able to isolate endogenous lung tumor-reactive CD8^+^ cells from KP.SIY lung tumor-bearing mice on days 7, 10, 13, and 16 post-tumor cell inoculation using PE-labeled, SIY-loaded MHC-I tetramers (Fig. 4a). Based on Klrg1 and IL-7Rα surface expression, these lung tumor-reactive CD8^+^ cells were segregated into a T_eff_ subset (Klrg1^hi^IL-7Rα^lo^) and a T_ems_ subset (Klrg1^lo^IL-7Rα^hi^) [26]. As a negative control, naïve CD8^+^ cells (CD44^lo^CD62L^hi^) were isolated on day 0 immediately prior to tumor inoculation. Consistent with our foregoing in silico analysis, *Zeb2* gene expression was most dramatically induced in the lung tumor-reactive T_eff_ compartment and was lowest in the lung tumor-reactive T_ems_ compartment, with negligible expression in naïve CD8^+^ cells (Fig. 4b). In our second KP.SIY model, TCR_2C_^+^CD8^+^ cells, which recognize the SIY-K^b^ ligand [24, 25], were adoptively transferred into KP.SIY lung tumor-bearing mice. Lung tumor-reactive TCR_2C_^+^CD8^+^ donor cells were sorted into T_eff_ and T_ems_ subsets on days 7, 10, 13, and 16 post-tumor cell inoculation. Similar to the first model, *Zeb2* gene expression was dramatically induced in the lung tumor-reactive T_eff_ compartment with minimal expression in the lung tumor-reactive T_ems_ compartment and naïve CD8^+^ cells (Fig. 4c).

Ectopic ZEB2 overexpression is cytotoxic to activated CD8^+^ cells [17], so Zeb2 gain-of-function studies cannot be practically employed here. Therefore, we constructed a Zeb2 conditional knock-out model wherein *Zeb2* was selectively deleted from activated CD8^+^ cells. As CD8^+^ lymphocytes upregulate Granzyme B (*Gzmb*) transcription upon activation [27], this was accomplished through crossing *Zeb2*-floxed (*Zeb2*^fl/fl^) mice to *Gzmb*-Cre mice to create *Zeb2*^fl/fl^;*Gzmb*-Cre (*Zeb2*^−/−^) mice. In both KP.SIY models, *Zeb2* knockout significantly suppressed the number of lung tumor-reactive CD8^+^ cells on days 7, 10, 13, and 16 post-tumor cell inoculation (Fig. 4d, e). In both KP.SIY models, *Zeb2* knockout significantly suppressed the proportion of lung tumor-reactive T_eff_ cells in the spleen on day 7 post-tumor cell inoculation (Fig. 4f, g). As *Zeb2*^fl/fl^ and *Zeb2*^−/−^ mice possessed differing proportions of lung tumor-reactive T_eff_ cells, we isolated lung tumor-reactive TCR_2C_^+^CD8^+^ donor T_eff_ cells from each genotype in order to directly compare gene expression within lung tumor-reactive T_eff_ cells. Notably, *Zeb2* knockout downregulated expression of T_eff_-signature genes (i.e., *Ccl3*, *Cx3cr1*, *Gzma*, *Gzmb*, *Klra3*, *Klrg1*, *Prdm1*, and *S1pr5* [17, 18]) in lung tumor-reactive TCR_2C_^+^CD8^+^ donor T_eff_ cells (Fig. 4h). In sum, these findings suggest that Zeb2 stimulates lung tumor-reactive T_eff_ cell differentiation.

### ZEB2 acts downstream of the transcription factor T-bet to stimulate lung tumor-reactive T_eff_ cell differentiation

**Fig. 5:**
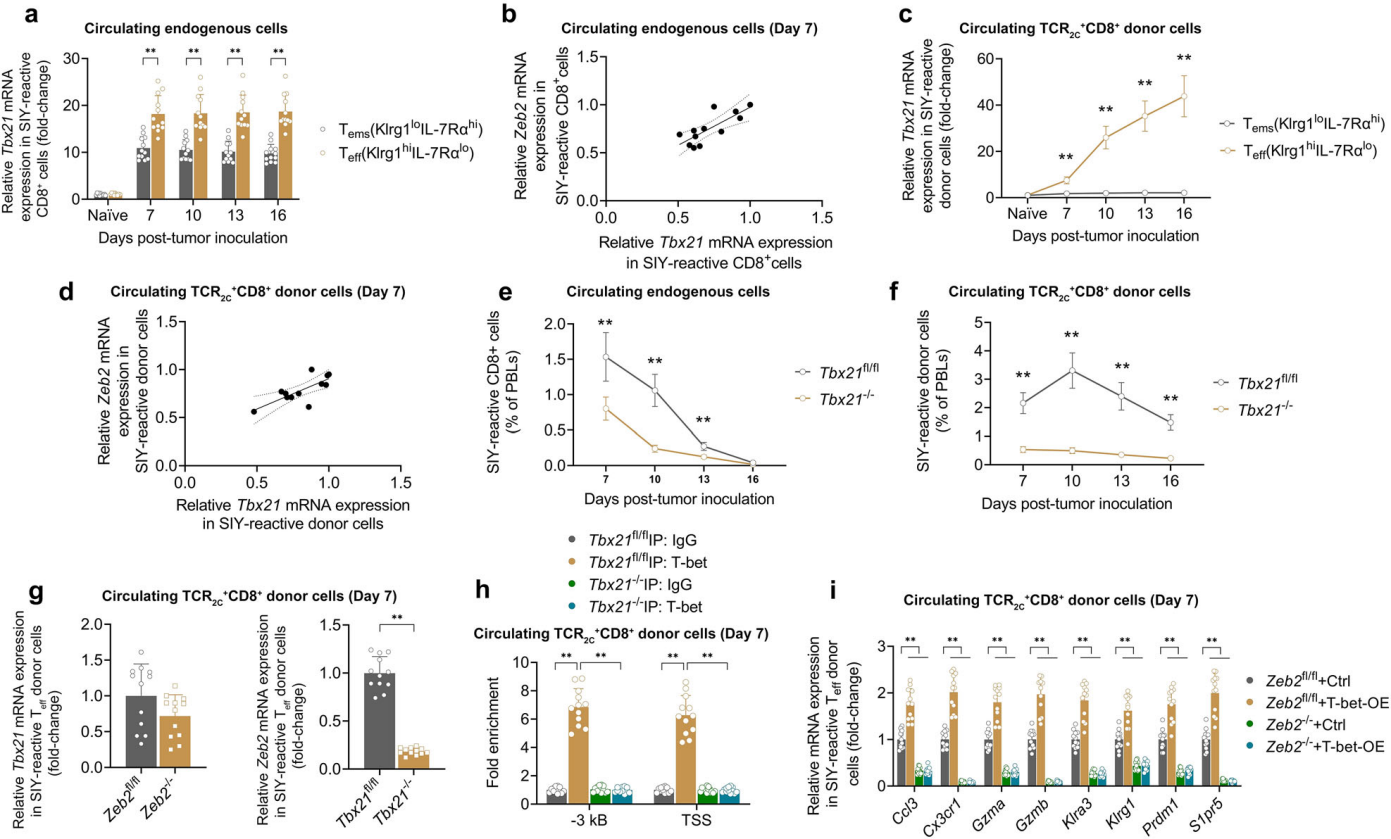
ZEB2 acts downstream of the transcription factor T-bet to stimulate lung tumor-reactive T_eff_ cell differentiation. **a**, **c** qPCR analysis of *Tbx21* mRNA expression in purified naïve CD8^+^ cells (CD44^lo^CD62L^hi^), SIY-reactive T_eff_ CD8^+^ cells (Klrg1^hi^IL-7Rα^lo^), or SIY-reactive T_ems_ CD8^+^ cells (Klrg1^lo^IL-7Rα^hi^) on the indicated days post-tumor inoculation in WT mice using the **a** endogenous CD8^+^ model and the (**c**) TCR_2C_^+^CD8^+^ adoptive transfer model. **b**, **d** Correlation analysis of *Tbx21* mRNA expression and *Zeb2* mRNA expression in SIY-reactive T_eff_ cells on days 7 post-tumor inoculation in WT mice using the (**b**) endogenous CD8^+^ model and the (**d**) TCR_2C_^+^CD8^+^ adoptive transfer model. **e**, **f** The percentage of SIY-reactive CD8^+^ cells on the indicated days post-tumor inoculation in *Tbx21*-floxed (*Tbx21*^fl/fl^) mice and *Tbx21*^fl/fl^; *Gzmb*-Cre (*Tbx21*^−/−^) mice using the (**e**) endogenous CD8^+^ model and the (**f**) TCR_2C_^+^CD8^+^ adoptive transfer model. **g**, **h** Analyses performed in the TCR_2C_^+^CD8^+^ adoptive transfer model on day 7 post-tumor inoculation. **g** (left) qPCR analysis of *Tbx21* mRNA expression in SIY-reactive TCR_2C_^+^CD8^+^ T_eff_ donor cells derived from *Zeb2*-floxed (*Zeb2*^fl/fl^) and *Zeb2*^fl/fl^; *Gzmb*-Cre (*Zeb2*^−/−^) mice; (right) qPCR analysis of *Zeb2* mRNA expression in SIY-reactive TCR_2C_^+^CD8^+^ T_eff_ donor cells derived from *Tbx21*^fl/fl^ and *Tbx21*^−/−^ mice. **h** ChIP analysis performed with an anti-T-bet or IgG control antibody at the 3′ UTR (−3 kB) and transcription start site (TSS) in SIY-reactive TCR_2C_^+^CD8^+^ T_eff_ donor cells derived from *Tbx21*^fl/fl^ and *Tbx21*^−/−^ mice. **i** Tumor-reactive TCR_2C_^+^CD8^+^ donor cells derived from *Zeb2*^fl/fl^ and *Zeb2*^−/−^ mice were spin-transduced with a retroviral T-bet overexpression vector (T-bet-OE) or empty control vector (Ctrl) and introduced to recipient tumor-bearing mice. qPCR analysis of T_eff_-signature genes in SIY-reactive TCR_2C_^+^CD8^+^ T_eff_ cells on day 7 post-tumor inoculation. Data depicts means ± SDs from *n* = 12 mice/group. *P < 0.05; **P < 0.01 [**a**–**f** two-way ANOVA, **g** *t*-test, **h**, **i** one-way ANOVA].

The transcription factor TBX21 (T-bet) is a direct transcriptional activator of ZEB2 in CD8^+^ cells [17, 18]. Consistent with *Zeb2* gene expression, *Tbx21* gene expression was most dramatically induced in the T_eff_ compartment and was lowest in the T_ems_ compartment of lung tumor-reactive CD8^+^ cells from KP.SIY lung tumor-bearing mice (Fig. 5a). Correlation analysis revealed a strong statistical correlation between *Tbx21* gene expression and *Zeb2* gene expression in T_eff_ cells (Fig. 5b). A similar pattern of findings was discovered with lung tumor-reactive TCR_2C_^+^CD8^+^ donor T_eff_ cells (Fig. 5c, d). Employing *Tbx21*-floxed (*Tbx21*^fl/fl^) mice to *Gzmb*-Cre mice to create *Tbx21*^fl/fl^;*Gzmb*-Cre (*Tbx21*^−/−^) mice, *Tbx21* knockout significantly suppressed the proportion of lung tumor-reactive T_eff_ cells in the spleen on day 7 post-tumor cell inoculation in both KP.SIY models (Fig. 5e, f).

As *Zeb2* knockout did not significantly impact *Tbx21* gene expression, while *Tbx21* knockout significantly downregulated *Zeb2* gene expression, in lung tumor-reactive TCR_2C_^+^CD8^+^ donor T_eff_ cells (Fig. 5g), we postulated that Zeb2 functions downstream of T-bet in lung tumor-reactive T_eff_ cells. ChIP-qPCR in *Tbx21*^fl/fl^ and *Tbx21*^−/−^ lung tumor-reactive TCR_2C_^+^CD8^+^ donor T_eff_ cells revealed that T-bet binds directly to the *Zeb2* promoter (Fig. 5h). To demonstrate T-bet’s dependency upon Zeb2, a *Tbx21*-expressing retrovirus was employed to overexpress the T-bet protein in *Zeb2*^fl/fl^ and *Zeb2*^−/−^ lung tumor-reactive TCR_2C_^+^CD8^+^ donor T_eff_ cells, which were then introduced to KP.SIY lung tumor-bearing mice. We isolated SIY-reactive TCR_2C_^+^CD8^+^ donor T_eff_ cells from each mouse cohort to directly compare T_eff_-signature gene expression. T-bet overexpression in *Zeb2*^fl/fl^ T_eff_ cells stimulated expression of all T_eff_-signature genes (Fig. 5i). In contrast, T-bet overexpression in *Zeb2*^−/−^ T_eff_ cells did not significantly impact expression of any T_eff_-signature genes. As T-bet displayed dependency upon Zeb2 in upregulating T_eff_-signature genes in lung tumor-reactive T_eff_ cells, Zeb2 appears to act downstream of T-bet to stimulate lung tumor-reactive T_eff_ cell differentiation.

### The T-bet/ZEB2 axis displays immunotherapeutic effects on KP.SIY lung tumors independent of ICB therapy

**Fig. 6:**
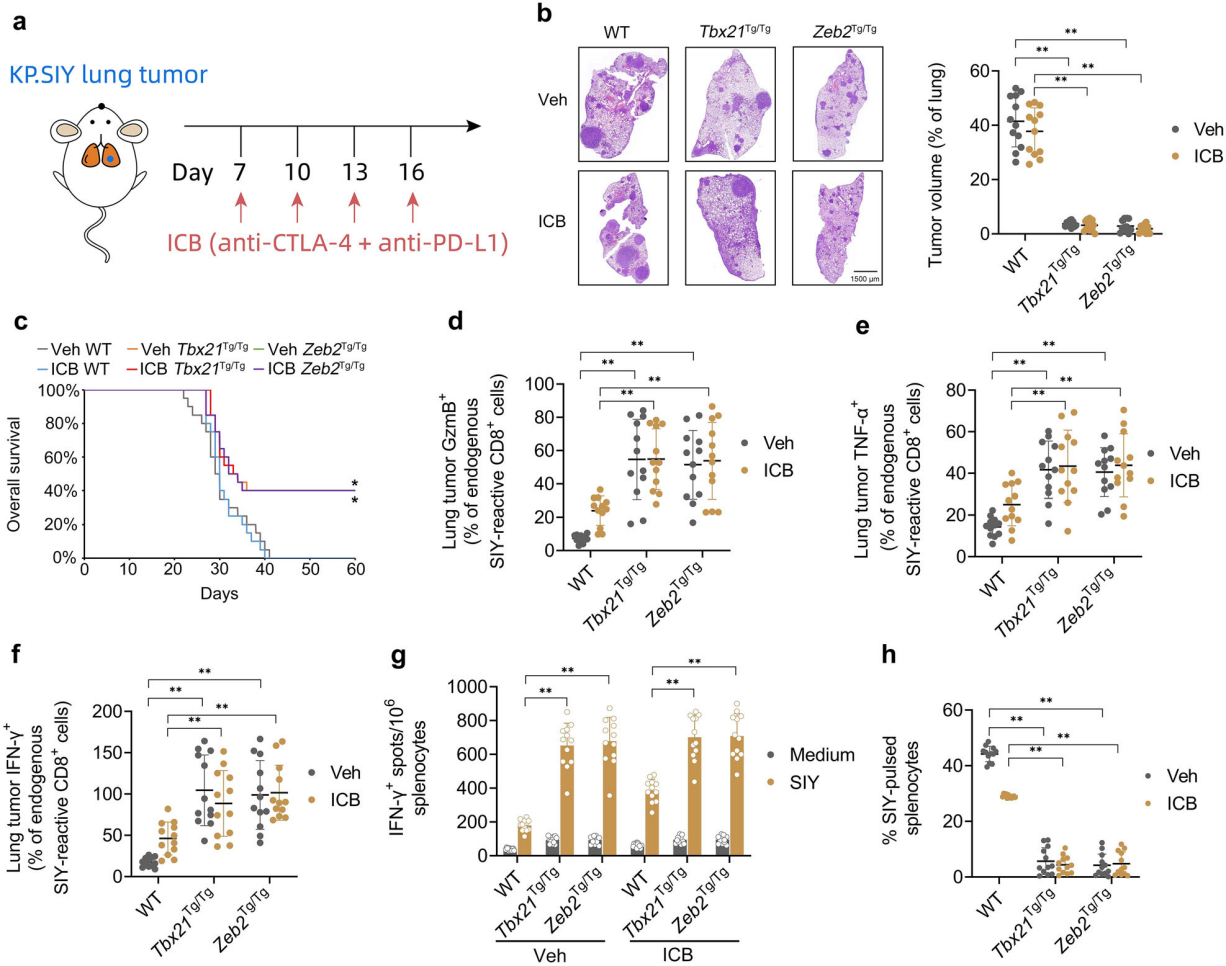
The T-bet/ZEB2 axis displays immunotherapeutic effects on KP.SIY lung tumors independent of ICB therapy. **a** Schematic of the ICB study. WT, *Tbx21*^Tg/Tg^, or *Zeb2* ^Tg/Tg^ mice inoculated with KP.SIY lung tumors received intraperitoneal (i.p.) injections of either vehicle control or immune checkpoint blockade (ICB) on days 7, 10, 13, and 16 post-tumor inoculation. Each i.p. ICB injection contained 100 mg anti-PD-L1 and 100 mg anti-CTLA-4. **b** Representative H&E images of lung tumor burden and quantification of the percentage of tumor area per lung lobe assessed on day 21 post-tumor inoculation. **c** Survival analysis of mice inoculated with KP.SIY lung tumors. **d–f** Analysis of (**d**) GzmB, **e** TNF-α, and **f** IFN-γ expression on endogenous SIY-reactive CD8^+^ cells in KP.SIY tumor-bearing mice on day 14 after tumor inoculation. **g** IFN-γ ELISpot assay of IFN-γ producing splenocytes from KP.SIY tumor-bearing mice on day 14 post-tumor inoculation. **h** To assay in vivo cytotoxicity, mice inoculated with KP.SIY lung tumors were challenged intravenously with SIY-pulsed CFSE^lo^ or unpulsed CFSE^hi^ splenocytes on day 14 post-tumor inoculation. Four hours following splenocyte challenge, mouse spleens were analyzed. Data depicts means ± SDs or medians ± ranges from *n* = 12 mice/group. *P < 0.05; **P < 0.01 [**b**, **d–h** two-way ANOVA, **c** log-rank test]. The figure is created using elements from BioRender.com.

Given that the T-bet/ZEB2 axis stimulates lung tumor-reactive T_eff_ cell differentiation, we hypothesized that T-bet or Zeb2 overexpression should display immunotherapeutic effects on KP.SIY lung tumors. WT, *Tbx21*^Tg/Tg^, or *Zeb2* ^Tg/Tg^ KP.SIY lung tumor-bearing mice were treated with vehicle or ICB on days 7, 10, 13, and 16 of tumor growth and monitored daily for survival (Fig. 6a). ICB alone produced no significant effects on lung tumor burden or survival (Fig. 6b, c). However, both T-bet and Zeb2 overexpression significantly reduced lung tumor burden and significantly prolonged survival tumors independent of ICB. Both T-bet and Zeb2 overexpression significantly enhanced the percentages of endogenous lung tumor-reactive GzmB^+^, TNF-α^+^, and IFN-γ^+^ CD8^+^ TILs independent of ICB (Fig. 6d–f). Moreover, both T-bet and Zeb2 overexpression significantly enhanced endogenous IFN-γ-producing, lung tumor-reactive splenocytes (Fig. 6g) and death of SIY-pulsed splenocytes (Fig. 6h) independent of ICB. In sum, this evidence suggests that the T-bet/ZEB2 axis displays immunotherapeutic effects on KP.SIY lung tumors independent of ICB therapy.

### The T-bet/ZEB2 axis is the primary mediator of the immunotherapeutic effects of IL-2 + IL-12 on KP.SIY lung tumors

ZEB2 overexpression plays a clear oncogenic role in early T-cell precursor leukemia (ETP-ALL) [28, 29]; therefore, direct application of T-bet or Zeb2 gain-of-function in CD8^+^ cells will likely be of little clinical utility. Horton et al. [14] has successfully employed the long-acting murine serum albumin (MSA)-cytokine fusion proteins MSA-IL2 and MSA-IL12 (alone or in combination) as anti-lung tumor cytokine immunotherapy in mice. We hypothesized that the T-bet/ZEB2 axis may be the primary mediator of the immunotherapeutic effects of MSA-IL2 + MSA-IL12 on KP.SIY lung tumors.

**Fig. 7:**
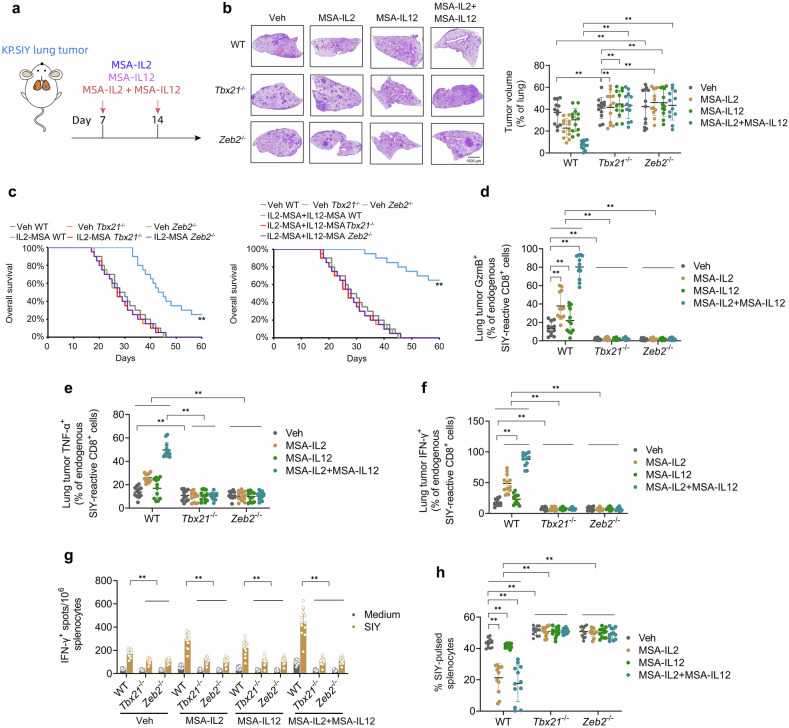
The T-bet/ZEB2 axis is the primary mediator of the immunotherapeutic effects of IL-2 + IL-12 on KP lung tumors. **a** Schematic of the murine serum albumin (MSA)-cytokine fusion study. WT, *Tbx21*^fl/fl^; *Gzmb*-Cre (*Tbx21*^−/−^), or *Zeb2*^fl/fl^; *Gzmb*-Cre (*Zeb2*^−/−^) mice inoculated with KP.SIY lung tumors received either vehicle control, IL-2-MSA, IL-12-MSA, or the combination on day 7 post-tumor inoculation. **b** Representative H&E images of lung tumor burden and quantification of the percentage of tumor area per lung lobe assessed on day 21 post-tumor inoculation. **c** Survival analysis of WT, *Tbx21*^−/−^, or *Zeb2*^−/−^ mice inoculated with KP.SIY lung tumors that received either vehicle control, IL-2-MSA, or IL-2-MSA + IL-12-MSA. **d–f** Analysis of (**d**) GzmB, **e** TNF-α, and **f** IFN-γ expression on endogenous SIY-reactive CD8^+^ cells in KP.SIY tumor-bearing mice on day 10 after tumor inoculation. **g** IFN-γ ELISpot assay of IFN-γ producing splenocytes from KP.SIY tumor-bearing mice on day 10 post-tumor inoculation. **h** To assay in vivo cytotoxicity, mice inoculated with KP.SIY lung tumors were challenged intravenously with SIY-pulsed CFSE^lo^ or unpulsed CFSE^hi^ splenocytes on day 10 post-tumor inoculation. Four hours following splenocyte challenge, mouse spleens were analyzed. Data depicts means ± SDs or medians ± ranges from *n* = 12 mice/group. *P < 0.05; **P < 0.01 [**b**, **d–h** two-way ANOVA, **c** log-rank test]. The figure is created using elements from BioRender.com.

WT, *Tbx21*^−/−^, or *Zeb2*^−/−^ KP.SIY lung tumor-bearing mice were treated with MSA-IL2 and MSA-IL12 (alone or in combination) on day 7 of tumor growth and monitored daily for survival (Fig. 7a). Both IL2-MSA and IL2-MSA + IL12-MSA significantly reduced lung tumor burden and significantly prolonged survival, with IL2-MSA + IL12-MSA producing more significant effects (Fig. 7b, c). IL12-MSA monotherapy did not significantly prolong survival (Supp. Fig. S4). These pro-survival effects of cytokine immunotherapy were dependent upon T-bet and Zeb2. IL2-MSA + IL12-MSA significantly enhanced the percentages of endogenous lung tumor-reactive GzmB^+^, TNF-α^+^, and IFN-γ^+^ CD8^+^ TILs in a T-bet/Zeb2-dependent manner (Fig. 7d–f). Moreover, IL2-MSA + IL12-MSA significantly enhanced endogenous IFN-γ-producing, lung tumor-reactive splenocytes (Fig. 7g) and death of SIY-pulsed splenocytes (Fig. 7h) in a T-bet/Zeb2-dependent manner. In sum, this evidence suggests that the T-bet/ZEB2 axis is the primary mediator of the immunotherapeutic effects of MSA-IL2 + MSA-IL12 on KP.SIY lung tumors.

### IL2-MSA + IL12-MSA immunotherapy operates through a parallel IL-2-FOXO1/IL-12-STAT4-mediated mechanism to promote T-bet/ZEB2 expression and lung tumor-reactive T_eff_ cell differentiation

**Fig. 8:**
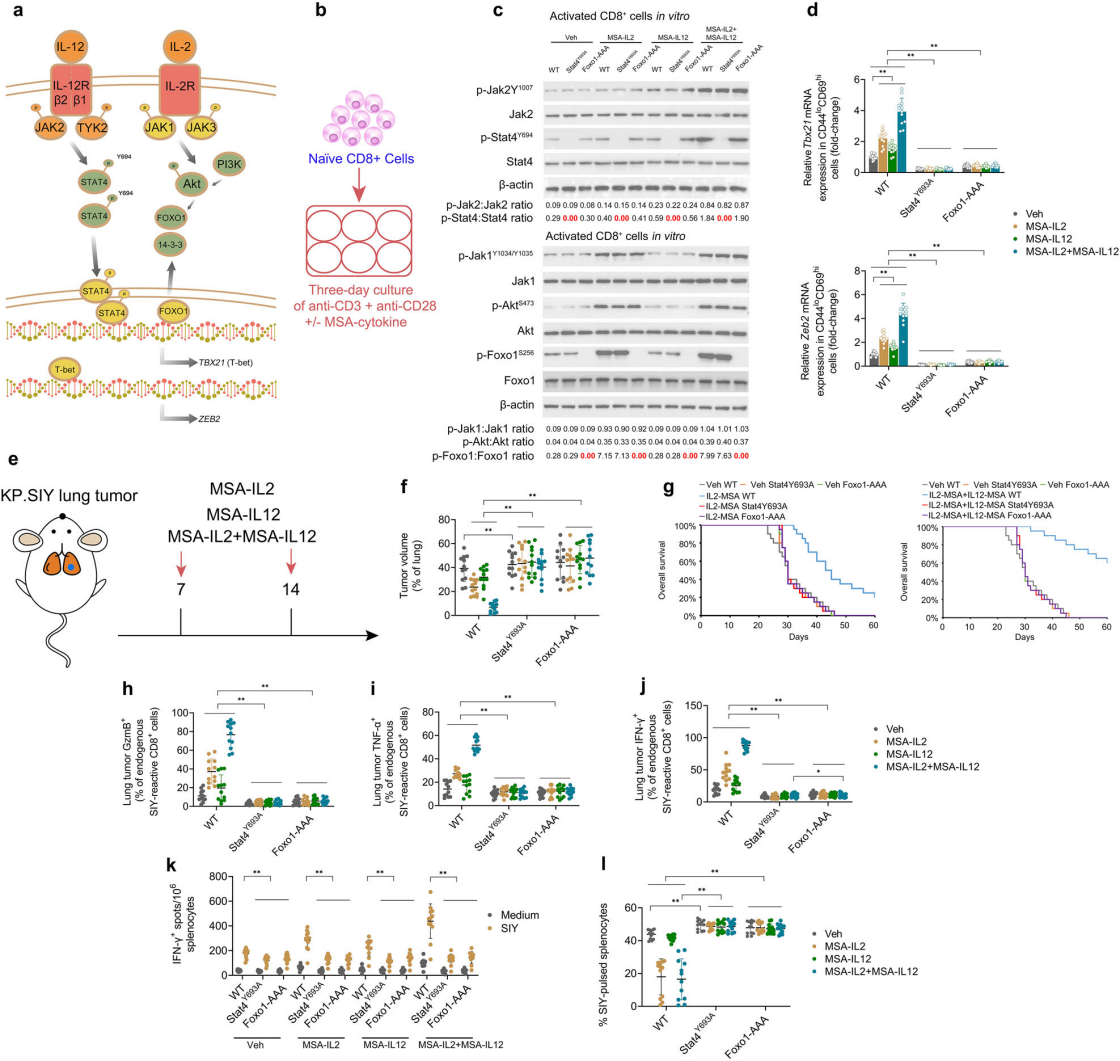
IL2 + IL12 immunotherapy operates through a parallel IL-2-FOXO1/IL-12-STAT4-mediated mechanism. **a** Schematic of the proposed parallel IL-2-Foxo1/IL-12-Stat4-mediated mechanism. **b** Naïve CD8^+^ cells isolated from WT mice, constitutive nuclear-localized Foxo1-triple alanine phosphorylation mutant mice (Foxo1-AAA), and phosphorylation-defective Stat4 mutant mice (Stat4^Y693A^) were activated ex vivo with anti-CD28 and anti-CD3 antibodies and treated with either vehicle control, IL-2-MSA, IL-12-MSA, or the combination for three days. **c** Western blotting analysis of Jak1/Akt signaling and Foxo1^S256^ phosphorylation as well as phosphorylation of Jak2^Y1007^ and Stat4^Y694^. Full, uncropped Western blots are provided in the Supplementary Material. **d** qPCR analysis of *Tbx21* mRNA expression and *Zeb2* mRNA expression. **e** Schematic of the murine serum albumin (MSA)-cytokine fusion study. WT, Foxo1-AAA, or Stat4^Y693A^ mice inoculated with KP.SIY lung tumors received either vehicle control, IL-2-MSA, IL-12-MSA, or the combination on day 7 post-tumor inoculation. **f** Quantification of the percentage of tumor area per lung lobe assessed on day 21 post-tumor inoculation. **g** Survival analysis of WT, Foxo1-AAA, or Stat4^Y693A^ mice inoculated with KP.SIY lung tumors that received either vehicle control, IL-2-MSA, or IL-2-MSA + IL-12-MSA. **h–j** Analysis of (**h**) GzmB, **i** TNF-α, and **j** IFN-γ expression on endogenous SIY-reactive CD8^+^ cells in KP.SIY tumor-bearing mice on day 10 after tumor inoculation. **k** IFN-γ ELISpot assay of IFN-γ producing splenocytes from KP.SIY tumor-bearing mice on day 10 post-tumor inoculation. **l** To assay in vivo cytotoxicity, mice inoculated with KP.SIY lung tumors were challenged intravenously with SIY-pulsed CFSE^lo^ or unpulsed CFSE^hi^ splenocytes on day 10 post-tumor inoculation. Four hours following splenocyte challenge, mouse spleens were analyzed. Data depicts means ± SDs or medians ± ranges from *n* = 12 mice/group. *P < 0.05; **P < 0.01 [**d**, **f**, **h–l** two-way ANOVA, **g** log-rank test]. The figure is created using elements from BioRender.com.

IL-12 induces Stat4 phosphorylation and downstream T-bet expression via a Stat-responsive enhancer on the *Tbx21* promoter in CD8^+^ cells [30]. Moreover, IL-2, via mTORC/Akt signaling, depresses Foxo1’s inhibition of *Tbx21* transactivation in CD8^+^ cells [31, 32]. Based on this evidence, we hypothesized that IL2-MSA + IL12-MSA immunotherapy operates through a parallel IL-2-Foxo1/IL-12-Stat4-mediated mechanism to promote T-bet and Zeb2 expression in CD8^+^ cells, with IL-2 depressing Foxo1’s blockade of IL-12/Stat4’s transactivation of *Tbx21* (Fig. 8a). To investigate this proposed mechanism in vitro, CD8^+^ cells were isolated from WT mice, constitutive nuclear-localized Foxo1-triple alanine phosphorylation mutant mice (Foxo1-AAA), and phosphorylation-defective Stat4 mutant mice (Stat4^Y693A^) were activated via anti-CD3 and anti-CD28, and subjected to vehicle, MSA-IL2, and MSA-IL12 (alone or in combination) (Fig. 8b). As expected, MSA-IL2-treated WT cells displayed enhanced Jak1/Akt signaling activation and Foxo1^S256^ phosphorylation, while MSA-IL12-treated WT cells displayed enhanced phosphorylation of Jak2^Y1007^ and Stat4^Y694^ (Fig. 8c). Under all conditions, the Foxo1-AAA mutant displayed strong Foxo1 nuclear localization, and the Stat4^Y693A^ mutant displayed no discernable Stat4^Y693^ phosphorylation. Notably, IL2-MSA + IL12-MSA dramatically enhanced expression of *Tbx21* and *Zeb2*, which were completely abrogated by Stat4^Y693A^ or Foxo1-AAA (Fig. 8d). WT, Foxo1-AAA, or Stat4^Y693A^ KP.SIY lung tumor-bearing mice were treated with MSA-IL2 and MSA-IL12 (alone or in combination) on day 7 of tumor growth and monitored daily for survival (Fig. 8e). Both IL2-MSA and IL2-MSA + IL12-MSA significantly reduced lung tumor burden and significantly prolonged survival, with IL2-MSA + IL12-MSA producing more significant effects (Fig. 8f, g). These pro-survival effects of cytokine immunotherapy were dependent upon Stat4 or Foxo1. IL12-MSA monotherapy did not significantly prolong survival (Supp. Fig. S5). IL2-MSA + IL12-MSA significantly enhanced the percentages of endogenous lung tumor-reactive GzmB^+^, TNF-α^+^, and IFN-γ^+^ CD8^+^ TILs in a Stat4 or Foxo1-dependent manner (Fig. 8h–j). Moreover, IL2-MSA + IL12-MSA significantly enhanced endogenous IFN-γ-producing, lung tumor-reactive splenocytes (Fig. 8k) and death of SIY-pulsed splenocytes (Fig. 8l) in a Stat4 or Foxo1-dependent manner. In sum, this evidence suggests that IL2-MSA + IL12-MSA immunotherapy operates through a parallel IL-2-FOXO1/IL-12-STAT4-mediated mechanism to promote T-bet/ZEB2 expression and lung tumor-reactive T_eff_ cell differentiation.

## Discussion

Consistent with Kissick and Amigorena’s multi-step activation model for CD8^+^TILs, our in silico findings revealed that naïve, non-exhausted CD8^+^ cells transition into (i) a cytotoxic effector trajectory (i.e., CX3CR1^+^T_ems_→T_eff_ cells) primarily localized in the circulation and adjacent normal lung tissue, (ii) a T_exh_ branch primarily localized within lung tumors, or (iii) a T_Ldys_ branch primarily localized within lung tumors. Given that fully-differentiated T_eff_ (and a subset of CX3CR1^+^T_ems_) express IFN-γ and exert cytotoxic activity in patients with lung cancer [8, 12, 13], we sought to identify master regulon(s) that may contribute to CD8^+^ differentiation along the cytotoxic effector trajectory in NSCLC tumors. Although several TFs have been generally associated with T_eff_ cell differentiation [33], our metaVIPER-based analyses revealed that the transcription factor ZEB2 may be a key driver of the cytotoxic effector trajectory in NSCLC tumors. Further examination of Zeb2 in two KP.SIY models revealed that *Zeb2* gene expression was dramatically induced in the lung tumor-reactive T_eff_ compartment with minimal expression in the lung tumor-reactive T_ems_ cells and naïve CD8^+^ cells. This consistent with previous research showing that ZEB2 expression correlates with the degree of T_eff_ cell differentiation in both human and mouse memory CD8^+^ cells [34, 35].

Similar to ZEB2, expression of the transcription factor T-bet correlates with the degree of T_eff_ cell differentiation [36, 37]. Using *Zeb2* and *Tbx21* conditional knock-out models, we discovered that Zeb2 functions downstream of T-bet in stimulating lung tumor-reactive T_eff_ cell differentiation. This consistent with previous research showing that ZEB2 is a direct transcriptional target of T-bet and that the ZEB2’s transcriptional program highly overlaps with T-bet’s transcriptional program in CD8^+^ cells [17, 18]. Further in vivo studies revealed that this T-bet/ZEB2 axis displays immunotherapeutic effects on KP.SIY lung tumors independent of ICB therapy and is the primary mediator of the immunotherapeutic effects of MSA-IL2 + MSA-IL12 on KP.SIY lung tumors. Similarly, the T-bet/ZEB2 axis has been shown to promote T_eff_ cell differentiation in response to primary and secondary infection [17, 18]. Mechanistically, this combination immunotherapy operates through a parallel IL-2-JAK1-FOXO1/IL-12-JAK2-STAT4-mediated mechanism to promote T-bet/ZEB2 expression and lung tumor-reactive Teff cell differentiation. As JAK is an upstream kinase for MEK1-ERK1/2 signaling in CD8^+^ cells [38], the current findings are consistent with Horton et al.’s work showing that IL2-MSA and IL12-MSA synergistically enhance ERK1/2 phosphorylation in activated CD8^+^ cells [14].

In conclusion, our findings highlight a previously unrecognized role of the transcription factor ZEB2 in supporting lung tumor-reactive T_eff_ cell differentiation and the anti-tumor efficacy of CD8^+^ TILs in NSCLC. Therefore, immunotherapeutic regimens that support ZEB2 activity in CD8^+^ cells may show particular promise in NSCLC patients.

## Methods

### In silico analyses of single-cell RNA sequencing (scRNAseq) data

Please see Supplementary Methods for descriptions of unsupervised clustering analyses, analysis of tissue preference, developmental trajectory modeling, phenotype scoring (i.e., exhaustion, cytotoxicity, and naiveness), network inference, and master regulon identification.

### Mice

Murine model studies were approved by our institution’s Institutional Animal Care and Use Committee (IACUC). All studies were conducted in accordance with IACUC guidelines and ARRIVE guidelines. All mice were housed in our institution’s animal facility under specific pathogen-free conditions. Euthanasia was performed via CO_2_ chamber followed by cervical dislocation.

Both male and female mice were used for experiments at 6–12 weeks of age. Sex- and age-matching were performed for all experiments. Wild-type (WT) C57BL/6 mice were obtained from the Jackson Laboratory (strain number 000664; RRID: IMSR_JAX:000664). *Zeb2*^flox/flox^ mice were originally generated by D. Huylebroeck (University of Leuven, Leuven, Belgium) [17]. Granzyme B-Cre (GzB-Cre+) mice from J. Jacobs (Emory University, GA, USA) and *Zeb2*^flox/flox^ mice were crossed to produce GzB-cre+; *Zeb2*^flox/flox^ (*Zeb2*^−/−^) mice and GzB-cre+; *Zeb2*^+/+^ or GzB-cre−; *Zeb2*^flox/flox^ (*Zeb2*^+/+^) mice. TCR_2C_ transgenic mice were additionally crossed with these *Zeb2*^−/−^ and *Zeb2*^+/+^ strains to generate TCR_2C_ *Zeb2*^−/−^ and TCR_2C_ *Zeb2*^+/+^ animals. TCR_2C_ chimeric mice were established via intravenously injecting approximately 50,000 TCR_2C_ CD8^+^ cells into WT C57BL/6 mice.

### Splenocyte isolation

Please see Supplementary Methods.

### Isolation of CD8^+^ tumor-infiltrating lymphocytes (CD8^+^TILs) from lung tumor tissue

Please see Supplementary Methods.

### Flow cytometry

Please see Supplementary Methods.

### Quantitative real-time PCR (qPCR)

Please see Supplementary Methods.

### Chromatin immunoprecipitation (ChIP)

Please see Supplementary Methods.

### Tumor cell culture and in vivo administration

The *Kras*^G12D/+^;*p53*^fl/fl^ tumor cell line exhibiting stable cerulean-SIYRYYGL expression (hereinafter termed “KP.SIY”) was established as in a prior report [9], with flow cytometry periodically being used to confirm cerulean expression. These tumor cells were cultured in DMEM (Gibco) containing 10% FBS (Atlanta Biologicals), 1× HEPES (Gibco), and 1% penicillin/streptomycin (Gibco) in a 37 °C/5% CO_2_ incubator. Trypsin (Gibco) was utilized to harvest cells, which were then rinsed twice using 1× PBS (Gibco), suspended in PBS, and intravenously administered to mice via the tail vein (2.5 × 10^5^/mouse). Survival analyses were then performed through the daily monitoring of these animals, euthanizing them as necessary based on the size of tumors or overall bodily status.

### Retroviral transduction

Please see Supplementary Methods.

### ELISpot

Please see Supplementary Methods.

### In vivo cytotoxicity assay

These experiments were performed as reported previously [9]. Briefly, WT splenocytes underwent ACK lysis treatment followed by a 1-h pulse with SIY peptide (0.2 mM) at 37 °C. These cells were then labeled using CFSE (0.5 mM), while control splenocytes that had not been pulsed with SIY were instead labeled with a higher CFSE concentration (5 mM) at 37 °C for 10 min. When labeling was complete, these CFSE-labeled control and SIY-pulsed splenocytes were mixed in equal amounts (1:1), and naïve mice or mice bearing KP.SIY lung tumors were intravenously injected with 10 × 10^6^ of each cell type on day 7 following tumor inoculation. Four hours following splenocyte injection, the spleens of these mice were collected and physically dissociated to prepare single-cell suspensions of the splenocytes therein. These cells were then subjected to live/dead staining and analyzed by flow cytometry to detect different CFSE^+^ populations.

### Immune checkpoint blockade (ICB) treatment

Mice were intraperitoneally injected with anti-PD-L1 monoclonal antibody (clone 10 F.9G2, Bio X Cell Cat# BE0101, RRID: AB_10949073) and anti-CTLA-4 monoclonal antibody (clone UC10-4F10-11, Bio X Cell Cat# BE0032, RRID: AB_1107598) on days 7, 10, 13, and 16 following tumor inoculation at a dose of 100 mg antibody per mouse for each injection.

### MSA-cytokine preparation and administration

After generating MSA-cytokine fusion proteins, they were subjected to size-exclusion chromatography-based purification and storage at 4 °C. For use, these cytokine fusions were warmed to room temperature, and for each treatment, mice were retro-orbitally injected with MSA-IL12 (1.42 × 10^−11^ mol) and/or MSA-IL2 (5.94 × 10^−10^ mol) per treatment. For experiments entailing the phenotyping of CD8^+^ cells, mice were dosed with these MSA-cytokine fusion preparations a single time on day 7 following tumor inoculation, with analyses then being conducted 3 days later (day 10 following tumor inoculation). For survival analyses, mice were dosed with MSA-IL12 and/or MSA-IL2 on day 7 and day 14 following tumor inoculation and then monitored for post-treatment survival.

### In vitro stimulation of CD8^+^ cells

An untreated, flat-bottom, 96-well plate was coated overnight at 4 °C with anti-CD3 (0.2 μg/mL; clone 145-2C11, BD Biosciences Cat# 564378, RRID: AB_2738779) and anti-CD28 (0.5 μg/mL; clone 37.51, BD Biosciences Cat# 553295, RRID: AB_394764) in PBS were used to coat the bottom of a flat-bottom, untreated, 96-well plate at 4 °C overnight. Plates were then rinsed using PBS and blocked at room temperature for a minimum of 30 min with RPMI (Gibco) supplemented with 10% FBS (Atlanta Biologicals), PBS, 1× β-mercaptoethanol (Gibco), and 1% penicillin/streptomycin (Gibco). An untouched CD8^+^ T cell isolation kit (Miltenyi Biotec) was used as directed to isolate CD8^+^ cells from the spleen of a naïve C57BL/6 mouse. These cells were then rinsed two times in PBS, followed by staining as directed using the CTV dye (Life Technologies). After labeling, these cells were added to the plates that had been coated with anti-CD3/anti-CD28 (10^5^ cells/well) in the same media as above in the presence of vehicle or MSA-cytokine fusion preparations as indicated. After incubating these cells for 3 days in a 37 °C/5% CO_2_ incubator, cells were subjected to analysis.

### Statistical analyses

R version 4.3.1 (R Project for Statistical Computing) was used to conduct bioinformatics analyses, and Prism 6 (GraphPad Software) was used to conduct all other statistical analyses. Sample sizes were equal to or exceeded those used in previous studies using the same animal models [9, 14]. Sample sizes as well as the definitions of statistical methods and measures for each experiment are provided in the corresponding figure legend. No animals were excluded from this study. Animals were allocated to experimental groups using a random number generator. Experiments were performed in a blinded manner; mice were coded and randomized by investigators not involved in experimental performance. Shapiro–Wilk’s test and Levene’s test were applied to assess data normality and homogeneity. Comparisons were made with Student’s *t*-tests, one-way ANOVAs with Tukey’s multiple comparison test, or two-way ANOVAs with Sidak’s multiple comparison test. Survival curves were constructed using the Kaplan–Meier method and statistically compared using the log-rank test. The indicated *P*-values (*P < 0.05; **P < 0.01) were deemed statistically significant.

### Ethics approval and consent to participate

All methods were performed in accordance with relevant guidelines and regulations. All animal experiments were approved by the IACUC of The Second Affiliated Hospital of Chongqing Medical University (IACUC-SAHCOMU-2025-0041). This study did not involve new human participants; only publicly available datasets were analyzed and no identifiable human images were used. Accordingly, IRB/ethics approval, informed consent to participate, and consent for publication are not applicable.

## Supplementary information

Uncropped Western Blots

Reproducibility Checklist

Supplementary Information

Supplementary File S1

Supplementary File S2

## Data Availability

The source code for the bioinformatics analyses can be accessed at https://github.com/NextGenSeek/CD8_NSCLC or by written request to the corresponding author.
