# Supplementary figures and images for: The transcription factor ZEB2 mediates the antitumor efficacy of tumor-infiltrating lymphocytes in non–small cell lung cancer
Source: Cell Death Dis. 2025 Nov 7;16(1):806. doi: 10.1038/s41419-025-08112-y (PMC12594841; doi:10.1038/s41419-025-08112-y)

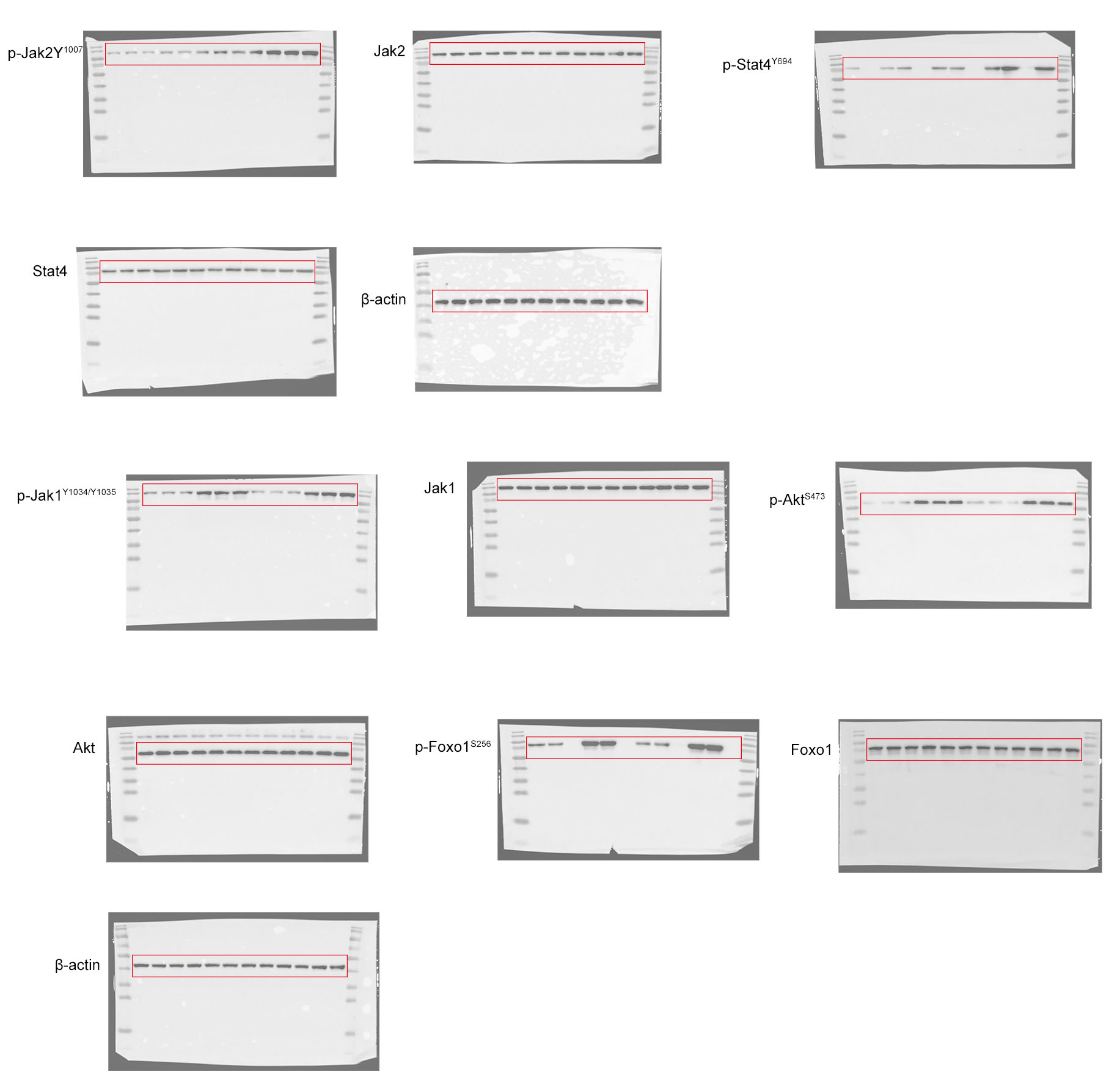

Supplement: Supplementary file 1 — Uncropped Western Blots [file 41419_2025_8112_MOESM1_ESM.jpg]
