# Supplementary material for: The transcription factor ZEB2 mediates the antitumor efficacy of tumor-infiltrating lymphocytes in non–small cell lung cancer
Source: Cell Death Dis. 2025 Nov 7;16(1):806. doi: 10.1038/s41419-025-08112-y (PMC12594841; doi:10.1038/s41419-025-08112-y)
Supplement: Supplementary file 3 — Supplementary Information [file 41419_2025_8112_MOESM3_ESM.pdf]

SUPPLEMENTARY INFORMATION FOR

The transcription factor ZEB2 mediates the antitumor efficacy of tumor-infiltrating lymphocytes in non–small cell lung cancer

Contents

SUPPLEMENTARY METHODS 2

Single-cell RNA sequencing (scRNAseq) dataset 2

Unsupervised clustering analyses 2

Analysis of tissue preference 2

Developmental trajectory modeling 3

Scoring of exhaustion, cytotoxicity, and naïveness 3

Network inference and master regulon identification 4

Splenocyte isolation 4

Isolation of $CD8^+$ tumor-infiltrating lymphocytes ($CD8^+$TILs) from lung tumor tissue 4

Flow cytometry 5

Quantitative real-time PCR (qPCR) 5

Chromatin immunoprecipitation (ChIP) 5

Retroviral transduction 6

ELISpot 6

SUPPLEMENTARY TABLES 8

Supplementary Table S1. Antibodies applied in flow cytometry studies. 8

Supplementary Table S2. Primers applied in qPCR studies. 8

SUPPLEMENTARY FIGURE LEGENDS 9

SUPPLEMENTARY FIGURES 10

Supplementary Figure S1 10

Supplementary Figure S2 11

Supplementary Figure S3 12

Supplementary Figure S4 13

Supplementary Figure S5 14

REFERENCES 15

SUPPLEMENTARY METHODS

Single-cell RNA sequencing (scRNAseq) dataset

We employed scRNAseq data from Guo et al.'s study that enrolled fourteen pathologically-diagnosed NSCLC patients, including eleven adenocarcinoma and three squamous cell carcinoma cases [1]. These patients were all free of any history of prior malignancies or autoimmunity, and none had undergone radiotherapy, chemotherapy, or antitumor medical treatment before their tumors had been resected. Guo et al.'s original publication provides the available details regarding the clinical characteristics of these patients. Guo et al. collected paired freshly-harvested NSCLC tumors and paracancerous tissue samples, as well as peripheral blood samples, for subsequent isolation of lymphocytes by fluorescence-activated cell sorting (FACS). The expression table employed in our analyses was comprised of Guo et al.'s normalized data on 12,415 genes from 11,769 cells.

Unsupervised clustering analyses

The $CD8^+$ cell expression matrix processing and clustering analyses were conducted as previously described [1]. Briefly, data from mucosal-associated invariant T (MAIT; $CD8^-C7^-SLC4A10^+$) cells and associated signature genes were excluded from the $CD8^+$ cell dataset, as they exhibit developmental processes and TCRs distinct from those of other $CD8^+$ cells. Clustering analyses were performed with the Seurat package. Initially, the top 1,500 genes were selected through the direct modeling of the mean-variance relationship to preserve the overall biological variability within the dataset. A t-SNE dimensionality reduction approach was then implemented, followed by the identification of clusters at a resolution of 0.8 and subclusters at a resolution of 0.4.

Analysis of tissue preference

Using chi-square testing, the ratio of observed cell numbers:random expectation cell numbers ($R_{O/E}$) was calculated for blood, normal lung tissue, and lung tumor tissue. A $R_{O/E}$ value of greater than unity represents

tissue enrichment. $R_{O/E}$ values were employed to adjust cell-sampling biases for each patient. The average $R_{O/E}$ values across all fourteen patients was employed as the summary measure.

Developmental trajectory modeling

Lineage differentiation trajectories among diverse populations of $CD8^+$ cells, utilizing CD8 cluster-associated genes as inputs, were constructed utilizing Monocle v2 as previously described [1]. Briefly, inferences regarding the differentiation trajectories of $CD8^+$ cells were then drawn using Monocle with the default parameters following dimensionality reduction and the ordering of cells. Patient-based trajectories were constructed by using the same processes, Monocle parameters, and signature genes for $CD8^+$ cells from each patient. The Monocle2 tool and diffusion maps were leveraged to infer pseudotime and developmental stages as previously described [2].

Scoring of exhaustion, cytotoxicity, and naïveness

Scoring of exhaustion, cytotoxicity, and naïveness was conducted as previously described [1]. Briefly, to compute exhaustion scores, DEGs were initially identified by comparing tumor-infiltrating $CD8^+$ cells between the exhausted group ($CD8^-C6^-LAYN^+$) and the non-exhausted group (all other cells) in the R limma package with a moderated *t*-test. To minimize the risk of false-positive results, a fold-change ≥ 4 and a Benjamini-Hochberg adjusted P-value<0.01 were established as thresholds. Ninety genes expressed at high levels in exhausted tumor-infiltrating $CD8^+$ cells were then selected, defining the exhaustion score for these cells as the average expression of these DEGs following their z-score transformation. Expression levels for each of these genes were calculated as $\log_2$(TPM+1). Average z-score transformed expression values for four well-established markers of naïveness (*CCR7*, *TCF7*, *LEF1*, and *SELL*) and twelve genes associated with cytotoxicity (*PRF1*, *NKG7*, *IFNG*, *GZMB*, *GZMA*, *GZMH*, *GNLY*, *KLRD1*, *KLRB1*, *KLRK1*, *CST7*, and *CTSW*) were similarly utilized to respectively calculate scores for naïveness and cytotoxicity of these $CD8^+$ cell populations. After assessing exhaustion, naïveness, and cytotoxicity scores of $CD8^+$ cell along the established trajectories,

relationships between these scores were fitted with Monocle components through a locally-weighted scatterplot smoothing (LOESS) regression approach.

Network inference and master regulon identification

Context-specific gene regulatory networks (GRNs) were inferred by utilizing the ARACNE algorithm from the RTN package as previously described [3]. ARACNE settings included 100 bootstraps and a data processing inequality tolerance of 0.01, utilizing curated transcription factor (TF) lists from a prior study [4]. Identification of master regulons was achieved with Califano et al.'s pipeline that was developed for master regulon inference from scRNA-seq datasets (https://github.com/califano-lab/single-cell-pipeline). Normalized enrichment score values derived from the metaVIPER (Virtual Inference of Protein activity by Enriched Regulon analysis) algorithm were employed to quantify the transcriptional activity as previously described [3].

Splenocyte isolation

Murine spleens were harvested and prepared into single-cell suspensions by masing them through a 70-μm filter. ACK lysis buffer (Gibco) was used to lyse erythrocytes in these lymphocyte samples for 3 min.

Isolation of CD8$^+$ tumor-infiltrating lymphocytes (CD8$^+$TILs) from lung tumor tissue

As previously described [6], fluorescently-labeled antibodies specific for CD45 (BD Biosciences Cat# 564279, RRID:AB_2651134) were retro-orbitally injected into tumor-bearing mice 3 min before these animals were euthanized to facilitate differentiation between circulating immune cell populations and non-circulating immune cell populations. Tumor-bearing lungs were resected from mice, weighed, and transferred into 5 mL of DMEM (Gibco) with enzymes from the human tumor dissociation kit (Miltenyi). The gentleMACS Octo Dissociator (Miltenyi) was used to dissociate tumors, after which a plunger from a 1-mL syringe was used to mash these digested lung tumors through a 70-μm filter to prepare single-cell suspensions. After rinsing these cells twice using PBS, they were layered onto Ficoll (GE) and spun for 30 min at 450 g, with brakes and acceleration set to the lowest setting. The cells at the Ficoll interface layer and the PBS fraction containing the

vast majority of viable immune cells were harvested, rinsed using PBS, and $CD8^+$TILs were isolated by flow cytometry as described below.

Flow cytometry

FACS staining buffer (cold PBS with 1% FBS and EDTA) was used to rinse cells, which were stained with eFluor 780 eBioscience Fixable Viability Dye (65-0865-14, Thermo Fisher Scientific) to allow for the identification of live/dead cells, together with SIY-loaded MHC-I tetramers labeled with PE (NIH Tetramer Core Facility) in a 50-μL volume containing fluorescently-conjugated antibodies (1:200). After a 20-min incubation on ice, cells were rinsed two times, incubated at room temperature for 30 min in Fixation/Permeabilization buffer (eBioscience), and suspended in staining buffer containing primary antibodies (Supp. Table S1). Precision Count Beads (BioLegend) were added to these samples and used as directed to obtain accurate absolute cell counts. A FACSAria III instrument (BD Biosciences) was used to collect flow cytometry data, which were then analyzed with FlowJo v10.5.3 (Tree Star). As previously described [6], $CD8^+$TILs were isolated by initially gating on cells with appropriate forward and side scatter, live status, $CD45^+$ status, CD45-IV^- status, $TCR\beta^+$ status, single cells, $CD4^-$ status, and $CD8^+$ status, with the PE-conjugated SIY-reactive MHC-I tetramer having been used to identify SIY-reactive $CD8^+$ cells. An SIY-pentamer (ProImmune) was included in the extracellular staining antibody mix at a 1:50 dilution.

Quantitative real-time PCR (qPCR)

An estimated 1.0×10^6 sorted cells were processed to isolate total RNA using the RNeasy kits and QIAshredder columns (QIAGEN) with QIAzol and RWT buffers, or using TRIzol (Life Technologies) and precipitation with ethanol. SSRTII (Life Technologies) was utilized to synthesize cDNA, after which the iTaq Universal SYBR Green Supermix (Bio-Rad Laboratories) and a Stratagene Mx3000P were used to conduct qRT-PCR analyses, utilizing the expression of *Rpl9* (L9) to calculate relative fold-change values. Sequences of the qPCR primers are provided in Supp. Table S2.

Chromatin immunoprecipitation (ChIP)

FACS was used to sort a total of 4.0×10^7 SIY-reactive $TCR_{2C}^+CD8^+$ T_{eff} cells from splenocytes on day 7 post-tumor inoculation. These cells were then fixed for 10 min using 1% formaldehyde, and glycine was added at a 0.125 M final concentration to quench this reaction. An appropriate buffer (1% SDS, 10 mM EDTA, pH 8, 50 mM Tris-HCL, pH 8) was then used to lyse these cells, followed by the sonication of these lysates to yield fragments approximately 250-750 bp in size. Then, immunoprecipitation reactions were performed by combining 10 μg of DNA and 5 μg of anti-T-bet (Bio X Cell Cat# BE0100, RRID: AB_10950173) or IgG control antibody (Bio-Rad Cat# PMP01X, RRID: AB_482871) at 4°C overnight with rotation. These solutions were then combined with 30 μL of protein F agarose beads (Cell Signaling Technology), followed by rotation at 4°C for a further 2 h. These beads were then spun down, rinsed, and eluted based on a protocol published by Cell Signaling Technology. The obtained DNA was subjected to reverse cross-linking at 65°C overnight, followed by RNase treatment at 37°C for 30 min and Proteinase K treatment at 55°C for 2 h. The Stratagene Brilliant II Sybr Green kit (Agilent Technologies) was used to conduct qPCR analyses with the *Tbx1* primers: 5′-ACCAAATCAGACCACGAGGA-3′ and 5′-ACTCTGTCTTGGCTGAACTGC-3′.

Retroviral transduction

To prepare virus-containing supernatants, human embryonic kidney 293T cells (ATCC) were transfected with a *Tbx21*-expressing pMXs-based retroviral vector (RRID: Addgene_21315) and pCL-ECO helper plasmid (RRID: Addgene_12371) using Fugene6 (Promega). 293T cells were confirmed mycoplasma-free by ATCC. $TCR_{2C}^+CD8^+$ donor mice were intravenously administered with KP.SIY lung tumor cells as described above. After one day, these donor mice were euthanized. Their splenocytes were harvested and spin-transduced with viral supernatants at 34°C for 90 min with polybrene (8 μg/mL). Transduced, lung tumor-reactive $TCR_{2C}^+CD8^+$ donor cells (1.0×10^5) were then intravenously transferred into recipient KP.SIY lung tumor-bearing mice. SIY-reactive $TCR_{2C}^+CD8^+$ donor T_{eff} cells were isolated from these recipient mice for qPCR analysis.

ELISpot

Anti-IFNγ (Bio X Cell Cat# BE0055, RRID: AB_1107694) was used to coat ELISpot plates (EMD Millipore) at 4°C overnight, after which these plates were blocked for 2 h at room temperature with DMEM containing 10% FBS, 1× HEPES, and 1% penicillin/streptomycin. On day 14 following KP.SIY lung tumor inoculation, spleens were harvested from experimental mice and prepared into a single-cell suspension by using the plunger of a 1-mL syringe to mash them through a 70-μm filter. ACK lysing buffer (500 μL; Gibco) was used to lyse erythrocytes for 2 min on ice, after which cold PBS was used to wash these splenocytes three times, followed by the analysis of 1×10^6 cells/well with or without SIY peptide (160 nM) or a positive control treatment consisting of a combination of PMA (100 ng/mL; Sigma-Aldrich) and ionomycin (1 μg/mL; Sigma-Aldrich). The following day, the mouse IFNγ ELISpot kit (BD Biosciences) was used to process these plates as directed.

SUPPLEMENTARY TABLES

Supplementary Table S1. Antibodies applied in flow cytometry studies.

Antibody	Supplier	Catalog number	RRID
Anti-CD4-BUV737, clone RM4-5	BD Biosciences	564933	RRID: AB_2732918
Anti-CD8a-BV605, clone 53.6-7	BioLegend	100744	RRID: AB_2562609
Anti-CD25-APC-Cy7, clone PC61	BD Biosciences	557658	RRID: AB_396773
Anti-CD45-BUV395, clone 30-F11	BD Horizon	564279	RRID: AB_2651134
Anti-GzmB-AF700, clone QA16A02	BioLegend	372222	RRID: AB_2728389
Anti-IFN-γ-BV650, clone XMG1.2	BD Biosciences	563854	RRID: AB_2738451
Anti-IL7R-PE, clone SB/199	BD Biosciences	552543	N/A
Anti-KLRG-APC, clone 2F1	BioLegend	138412	RRID: AB_10641560
Anti-TCRβ-BV711, clone H57-597	BioLegend	109207	N/A
Anti-TNFα-PE, clone MP6-XT22	BD Biosciences	554419	RRID: AB_395380)

Supplementary Table S2. Primers applied in qPCR studies.

Gene target	Forward sequence (5′→3′)	Reverse sequence (5′→3′)
Zeb2	GCAGTGAGCATCGAAGAGTACC	GGCAAAAGCATCTGGAGTTCCAG
Ccl3	ACTGCCTGCTGCTTCTCCTACA	ATGACACCTGGCTGGGAGCAAA
Cx3cr1	GAGCATCACTGACATCTACCTCC	AGAAGGCAGTCGTGAGCTTGCA
Gzma	GTGGTGGAAAGGACTCCTGCAA	GAGAGGAAAGTATAGACACCAGG
Gzmb	CAGGAGAAGACCCAGCAAGTCA	CTCACAGCTCTAGTCCTCTTGG
Klra3	GAGAACAGGACAGATGGGACAG	GCAGTTCGCTTTACATCCACTCC
Klrg1	CGAGGAATGGTAGCCACTGTTAC	CCGATCCAGTAAAAGTCCTGACC
Prdm1	AAGACGTTCGGTCAGCTCTCCA	CTGGCACTCATGTGGCTTCTCT
S1pr5	AGACTCCTCCAACAGCTTGCAG	TAGAGCTGCGATCCAAGGTTGG
Tbx21	ACCAAATCAGACCACGAGGA	ACTCTGTCTTGGCTGAACTGC

SUPPLEMENTARY FIGURE LEGENDS

Supplementary Figure S1. Deep scRNA-seq analyses of $CD8^+$ T-cells from treatment-naïve NSCLC patients. t-SNE projections of $CD8^+$ cells for each individual patient analyzed by cell cluster. Each dot represents one single cell and is color-coded by patient.

Supplementary Figure S2. Computational inference of the developmental trajectories of the $CD8^+$ clusters. Monocle-based inference displays the branched developmental trajectory of $CD8^+$ cells across two dimensions (naiveness and exhaustion). Each dot represents one single cell and is color-coded by patient.

Supplementary Figure S3. Identification of master regulon(s) that may contribute to $CD8^+$ differentiation along the cytotoxic effector trajectory. Bar charts of all metaVIPER-based regulons for each developmental branch. Regulons are charted in decreasing order of statistical significance.

Supplementary Figure S4. Survival analysis of WT, *Tbx21*$^{-/-}$, or *Zeb2*$^{-/-}$ mice inoculated with KP.SIY lung tumors that received either vehicle control or IL-12-MSA.

Supplementary Figure S5. Survival analysis of WT, Foxo1-AAA, or Stat4^{Y693A} mice inoculated with KP.SIY lung tumors that received either vehicle control or IL-12-MSA.

SUPPLEMENTARY FIGURES

Supplementary Figure S1

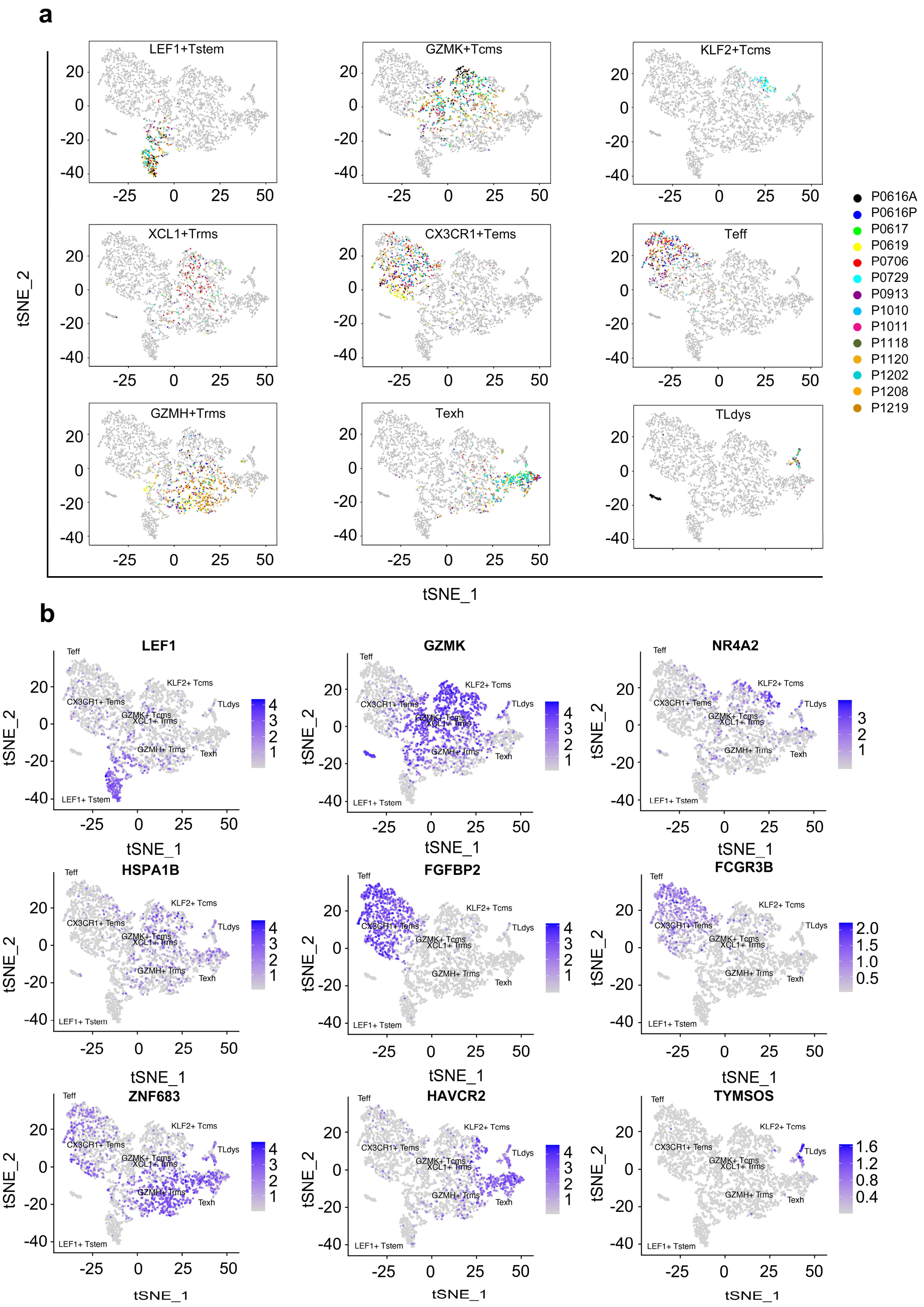

Supplementary Figure S2

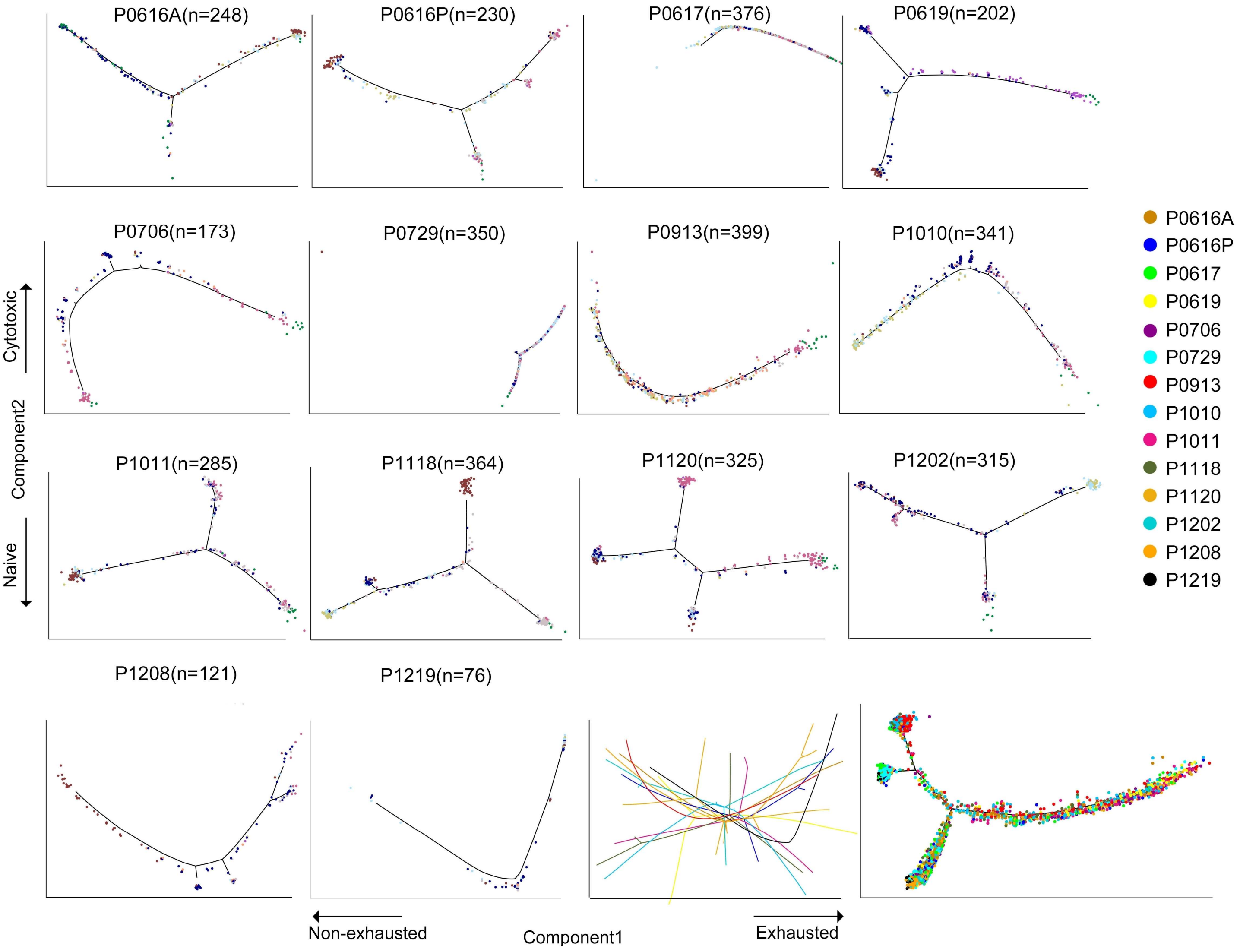

Supplementary Figure S3

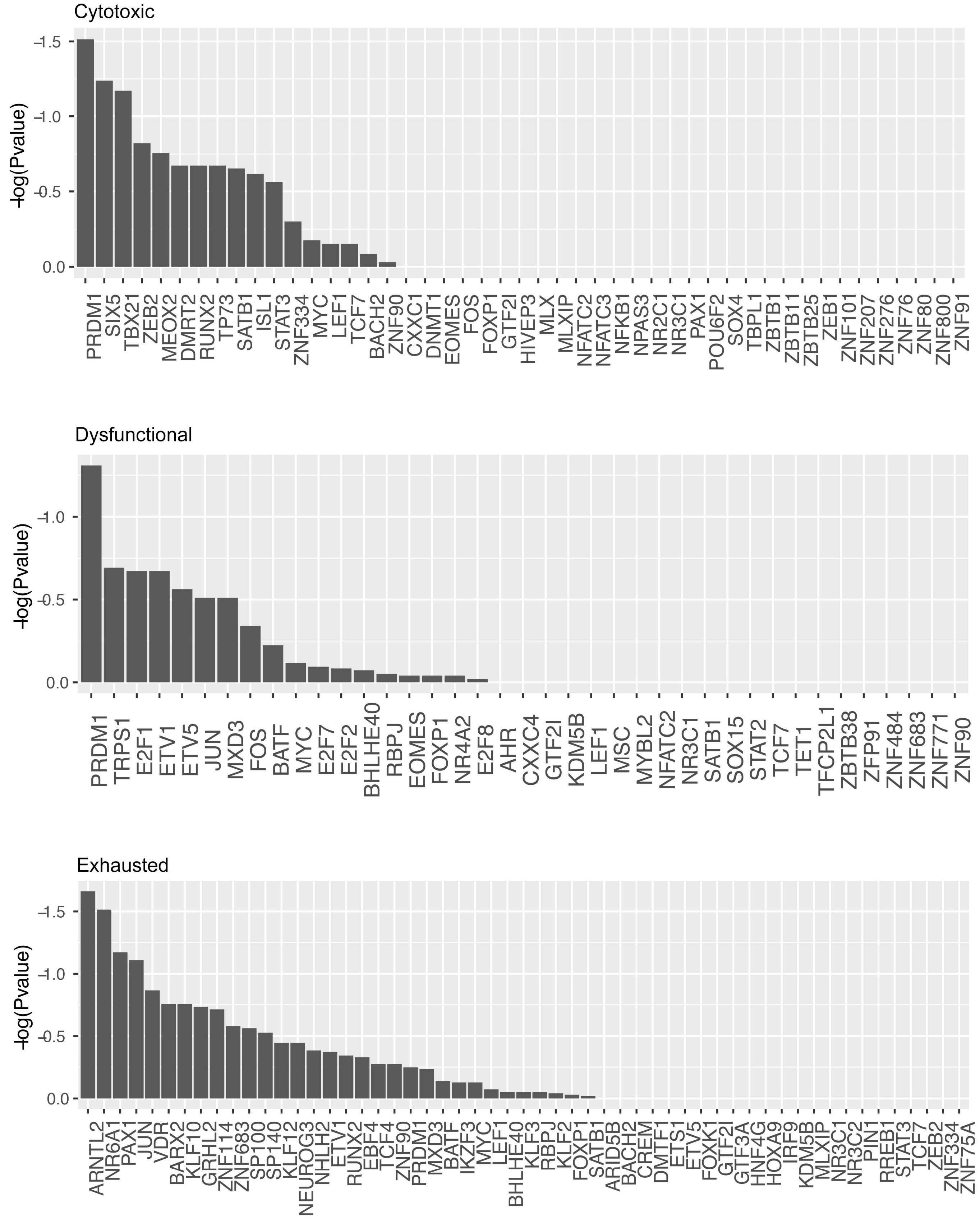

Supplementary Figure S4

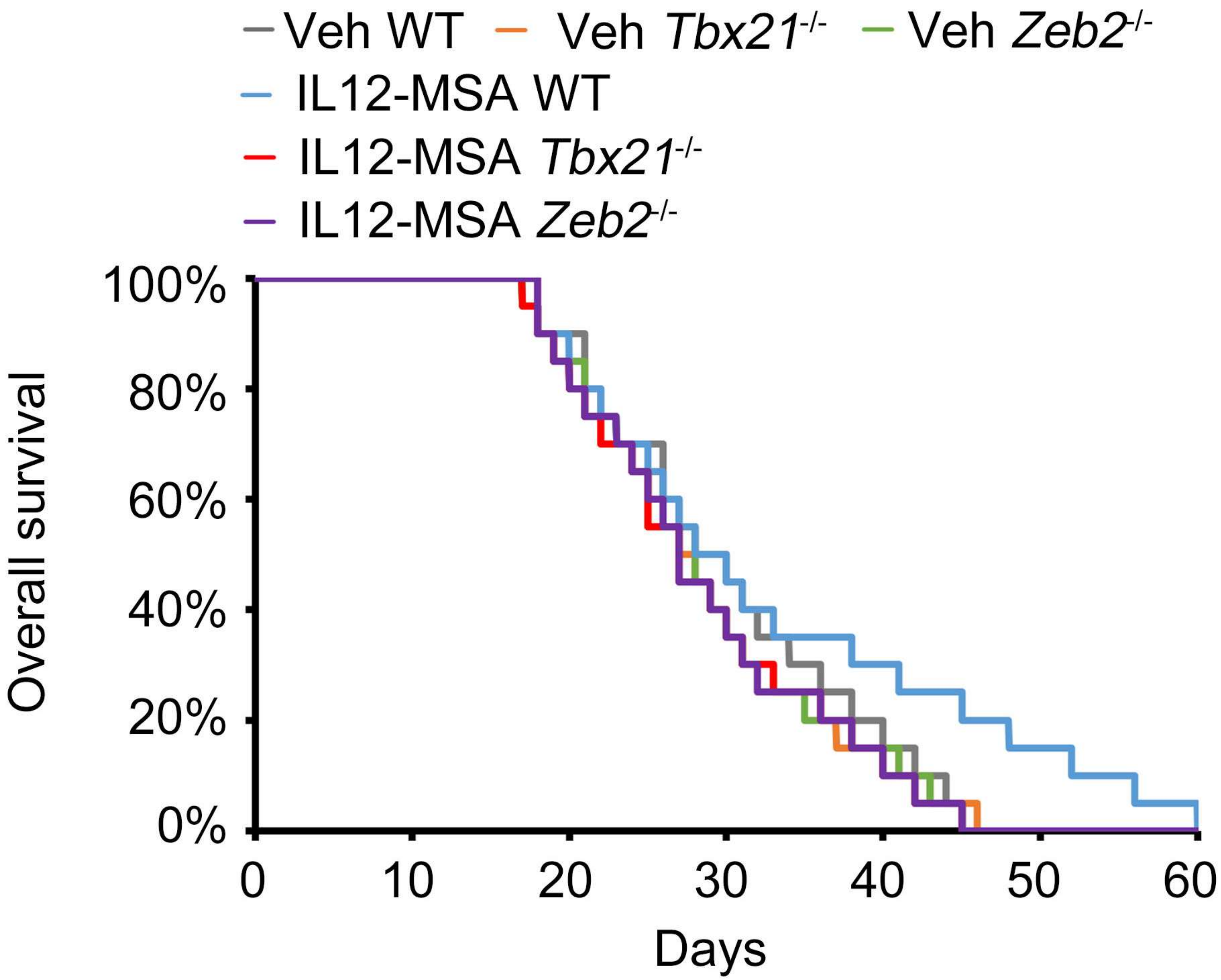

Supplementary Figure S5

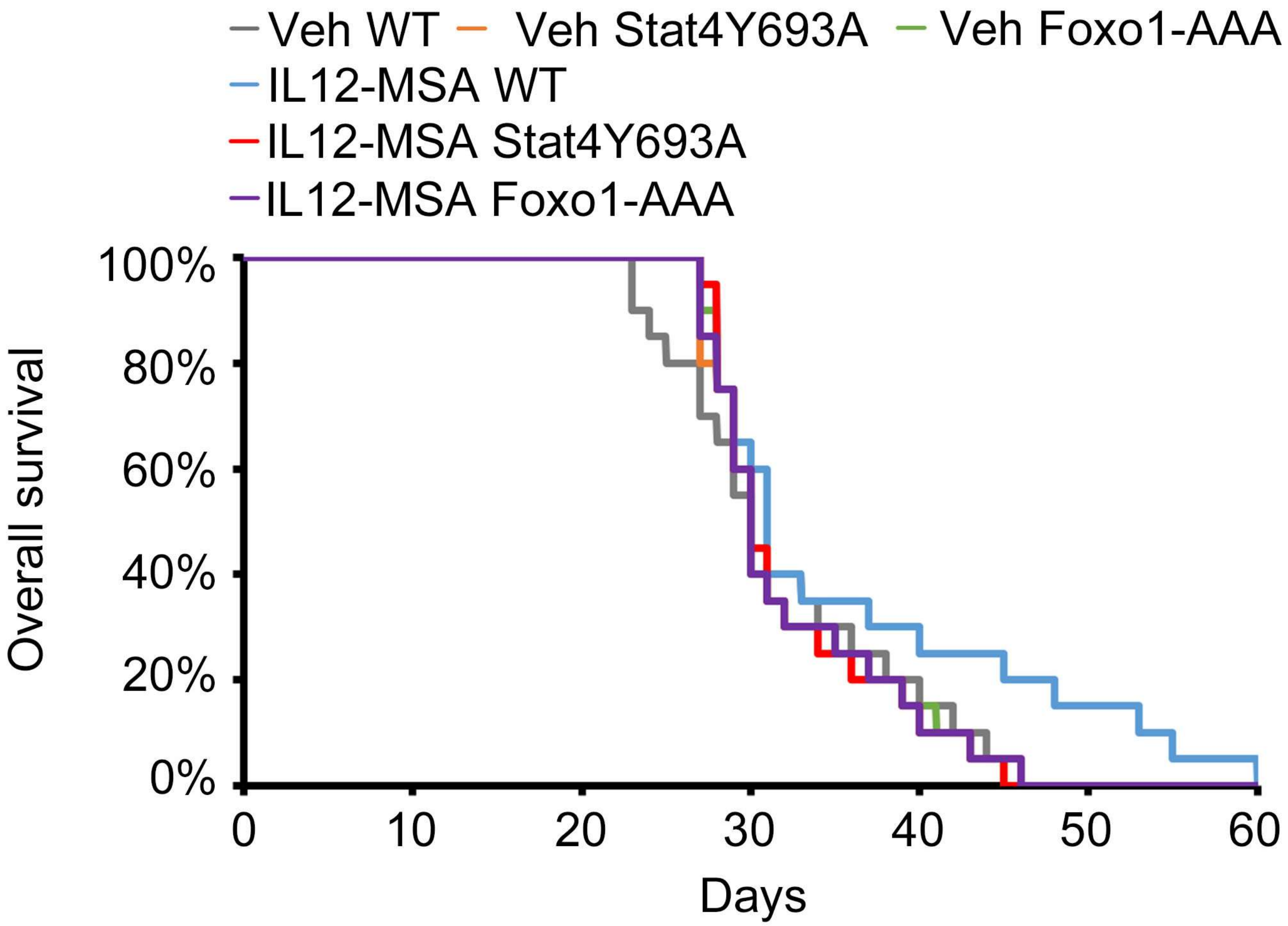

REFERENCES

1 Guo, X. *et al.* Global characterization of T cells in non-small-cell lung cancer by single-cell sequencing. *Nature medicine* **24**, 978-985 (2018).

2 Yan, K.-K. *et al.* Single-cell analysis identifies TCF4 and ID3 as a molecular switch of mammary epithelial stem cell differentiation. *bioRxiv*, 2020.2008. 2016.249854 (2020).

3 Lee, S. *et al.* Network inference analysis identifies SETDB1 as a key regulator for reverting colorectal cancer cells into differentiated normal-like cells. *Molecular Cancer Research* **18**, 118-129 (2020).

4 Garcia-Alonso, L., Holland, C. H., Ibrahim, M. M., Turei, D. & Saez-Rodriguez, J. Benchmark and integration of resources for the estimation of human transcription factor activities. *Genome research* **29**, 1363-1375 (2019).

5 Dominguez, C. X. *et al.* The transcription factors ZEB2 and T-bet cooperate to program cytotoxic T cell terminal differentiation in response to LCMV viral infection. *Journal of Experimental Medicine* **212**, 2041-2056 (2015).

6 Horton, B. L. *et al.* Lack of CD8+ T cell effector differentiation during priming mediates checkpoint blockade resistance in non–small cell lung cancer. *Science immunology* **6**, eabi8800 (2021).
